# Supplementary material for: Inhibition of mitochondrial complex I reverses NOTCH1-driven metabolic reprogramming in T-cell acute lymphoblastic leukemia
Source: Nat Commun. 2022 May 19;13:2801. doi: 10.1038/s41467-022-30396-3 (PMC9120040; doi:10.1038/s41467-022-30396-3)
Supplement: Supplementary file 1 — Supplementary Information [file 41467_2022_30396_MOESM1_ESM.pdf]

Inhibition of mitochondrial complex I reverses *NOTCH1*-driven metabolic reprogramming in T-cell acute lymphoblastic leukemia

Natalia Baran[1], Alessia Lodi[2], Yogesh Dhungana[3], Shelley Herbrich[1], Meghan Collins[2], Shannon Sweeney[2], Renu Pandey[2], Anna Skwarska[1], Shraddha Patel[1], Mathieu Tremblay[4], Vinitha Mary Kuruvilla[1], Antonio Cavazos[1], Mecit Kaplan[5], Marc O. Warmoes[6], Diogo Troggian Veiga[7], Ken Furudate[1,8], Shanti Rojas-Sutterin[4], Andre Haman[4], Yves Gareau[4], Anne Marinier[4], Helen Ma[1], Karine Harutyunyan[1], May Daher[5], Luciana Melo Garcia[5], Gheath Al-Atrash[5], Sujan Piya[1], Vivian Ruvolo[1], Wentao Yang[9], Sriram Saravanan Shanmugavelandy[10], Ningping Feng[11], Jason Gay[11], Di Du[6], Jun J. Yang[9], Fieke W. Hoff[1], Marcin Kaminski[12] , Katarzyna Tomczak[13], , R. Eric Davis[14], Daniel Herranz[15], Adolfo Ferrando[16], Elias J. Jabbour[1], M. Emilia Di Francesco[17], David T. Teachey[18], Terzah M. Horton[19], Steven Kornblau[1], Katayoun Rezvani[5], Guy Sauvageau[4], Mihai Gagea[20], Michael Andreeff[1], Koichi Takahashi[1], Joseph R. Marszalek[11], Philip L. Lorenzi[6], Jiyang Yu[21], Stefano Tiziani[2], Trang Hoang[4,22], Marina Konopleva[1*]

Supplementary Table 1. Panel of antibodies used for Western blotting.

Antibody	Cat. Number	Species	Company
Notch1 FL	3608s	rabbit	Cell Signaling
Notch1 cleaved	4147s	rabbit	Cell Signaling
p-Akt s473	4060s	rabbit	Cell Signaling
tAkt	2920s	mouse	Cell Signaling
p-4EBP1 s65	9456 s	rabbit	Cell Signaling
4EBP1	9452s	rabbit	Cell Signaling
p-eIF4E s209	9741s	rabbit	Cell Signaling
eIF4E	9724s	rabbit	Cell Signaling
p-S6 ser240/244	2215s	rabbit	Cell Signaling
S6	2217s	rabbit	Cell Signaling
p-PFKFB2 ser483	13064s	rabbit	Cell Signaling
PFKFB	13045s	rabbit	Cell Signaling
p-LDHA tyr10	8176s	rabbit	Cell Signaling
LDHA	2012 s	rabbit	Cell Signaling
Pyruvate dehydrogenase	3205s	rabbit	Cell Signaling
pLKB1 s428	3482s	rabbit	Cell Signaling
pAMPK t172	2535s	rabbit	Cell Signaling
AMPK	5832s	rabbit	Cell Signaling
pULK s555	5869s	rabbit	Cell Signaling
ULK1	4776s	rabbit	Cell Signaling
LC3A/B	4108s	rabbit	Cell Signaling
pH2AX s139	2577l	rabbit	Cell Signaling
H2AX	7631s	rabbit	Cell Signaling
c-myc	9402s	rabbit	Cell Signaling
pp-S6 kinase t389	97596s	rabbit	Cell Signaling
p-70 S6 kinase	9202	rabbit	Cell Signaling
Cleaved PARP	5625s	rabbit	Cell Signaling
PARP	9542s	rabbit	Cell Signaling
Cleaved caspase 3	9664L	rabbit	Cell Signaling
Caspase 3	9662s	rabbit	Cell Signaling
LKB1	ab15095	mouse	Abcam
mtTFA	ab47517	rabbit	Abcam
VDAC1	ab15895	rabbit	Abcam
Mitoprofile total oxphos human wb	ab110411	mouse	Abcam
GAC	ab156876	rabbit	Abcam
GAPDH	ab8245	mouse	Abcam
Tomm20	ab186735	mouse	Abcam
α-tubulin	ab7291	mouse	Abcam
β-Actin	A5441	mouse	SIGMA

Supplementary figures:

Supplementary Figure 1

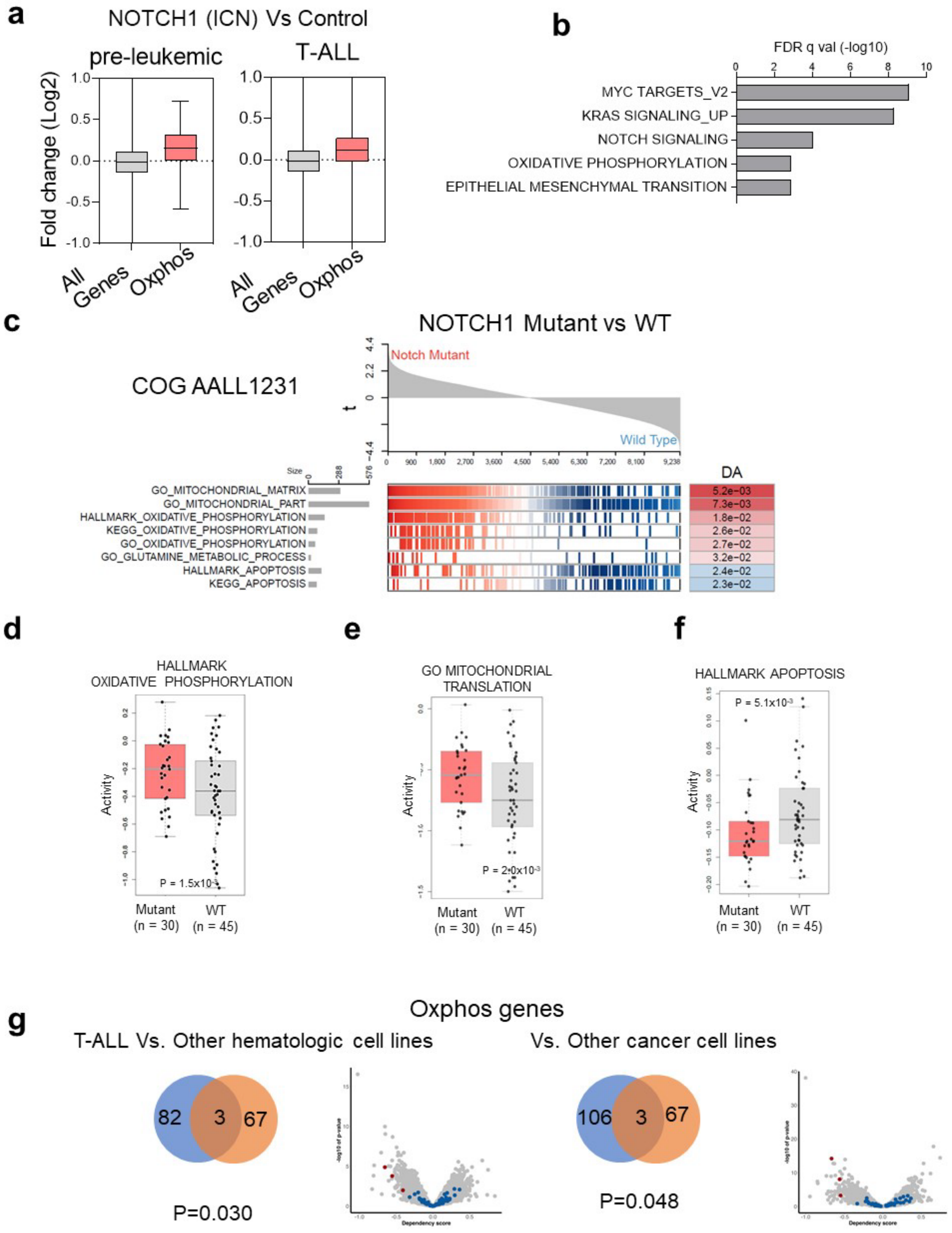

Supplementary Figure 1. OxPhos co-segregates with NOTCH1 gene in preleukemic and leukemic cell

a) Gene expression in murine CD4+CD8+ DP cells with or without expression of the *NOTCH1/ICN* oncogene (GSE12948) at the preleukemic stage (left panel) (n=70) or at the time of overt leukemia (T-ALL, right panel) (n=253). Data are shown as fold change of gene expression in *ICN*-expressing versus control (empty vector) DP cells (n=41646). The box plot presents the 25th to 75th percentiles with the median value highlighted and whiskers marking the minimum and maximum value. (Log2 transformed data; Wilcoxon rank sum test: $P = 10^{-8}$, left panel; $P = 8.6 \times 10^{-6}$, right panel).

b) Genes that were upregulated by the NOTCH1 oncogene (ICN) in $CD4^+CD8^+$ DP cells were analyzed for pathway enrichment (FDR ≤ 0.1, GSE12948). Shown are the FDR q values (−log10) for overlaps computed with the reference datasets in MSigDB.

c) Gene set enrichment analysis (GSEA) of gene set from AALL1231 trials (N=75), indicated enrichment of genes related to glutamine metabolism and mitochondrial metabolism as well as translation and downregulation of apoptosis-related genes in patients with NOTCH1 mutations (each vertical bar in x axis is gene rank in the pathway list and y axis represents running enrichment score).

d) The OxPhos gene signature was significantly enriched in T-ALL patients with NOTCH1 mutations in the TARGET cohort of 265 patients (b) and the COG AALL1231 cohort of 75 patients (f); two-sided t-test; The center line represents the median and whiskers represents maximum (Q3 + 1.5*IQR) and minimum value (Q1 + 1.5*IQR);

e) Patient samples with NOTCH1 mutations were characterized by significant enrichment of mitochondrial translation genes in both the TARGET (c) and COG AALL1231 (g) cohorts; two-sided t-test; The center line represents the median and whiskers represents maximum (Q3 + 1.5*IQR) and minimum value (Q1 + 1.5*IQR);

f) Gene expression analysis in the TARGET (d) and COG AALL1231 (h) cohorts showed lower enrichment of apoptosis-related genes in patients with NOTCH1 mutations than in those with wild-type NOTCH1. two-sided t-test; The center line represents the median and whiskers represents maximum (Q3 + 1.5*IQR) and minimum value (Q1 + 1.5*IQR);

g) Venn diagrams enriched by over representation analysis of differentially expressed genes, and Volcano plots showing cancer dependencies associated with OxPhos-related genes, graphed as P value (-log10, y-axis) against effect size (x-axis), comparing T-ALL versus other hematological malignancies (left) and T-ALL versus other cancers (right), two-sided t-test;

Supplementary Figure 2

a

	mouse #	DNA	Protein	Premature stop
Pre-leukemic	26056	Wt	Wt	NA
Pre-leukemic	26058	Wt	Wt	NA
Pre-leukemic	26059	Wt	Wt	NA
T-ALL	2398	7200-7201 delCA	frameshift AA 2400	in PEST domain
T-ALL	3303	7174insAT	frameshift AA 2391	before PEST domain
T-ALL	27259	6696del_insTCC	frameshift AA 2232	before PEST domain

b

	MS5-DL4		MS5	
Genotype	Maximum inhibition (%)	EC50 (nM)	Maximum inhibition (%)	EC50 (nM)
LMO1^{tg}	97 ± 8	56 ± 5	NA	N.A.
SCL^{tg}*LMO1*^{tg}	76 ± 6	39 ± 2	N.A.	N.A.
NOTCH1^{tg}	83 ± 12	53 ± 7	71 ± 8	52 ± 5
LMO1^{tg}*NOTCH1*^{tg}	95 ± 5	36 ± 3	91 ± 13	90 ± 3
SCL^{tg}*LMO1*^{tg}*NOTCH1*^{tg}	88 ± 8	50 ± 3	78 ± 7	52 ± 5

c

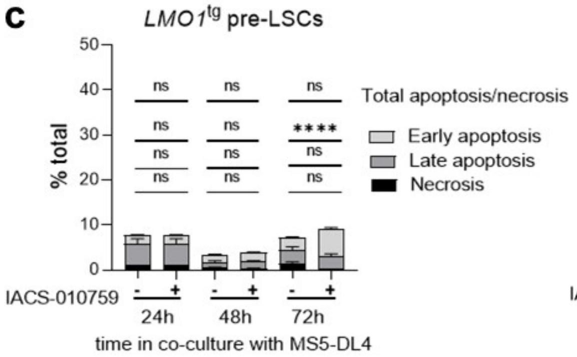

d

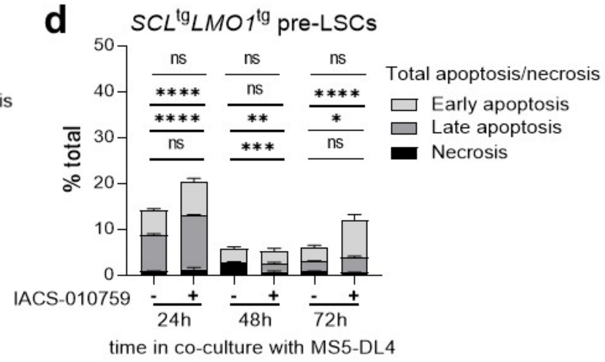

e

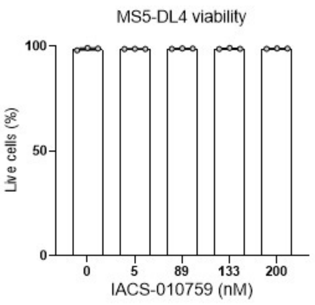

f

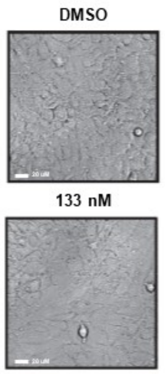

Supplementary Figure 2. NOTCH1 activation affects the response to OxPhos-inhibition of primary pre-LSCs.

a) Notch1 gene status in *SCL*^{tg}*LMO1*^{tg} preleukemic and leukemic thymocytes. *Notch1* gene activating mutations and their impact on protein sequences are shown. PEST, protein degradation domain;

b) Dose-response analysis of pre-LSCs to IACS-010759. Dose-response curves shown in Figures 2C were analyzed by non-linear curve fitting, to compute the maximum inhibition and the EC50 for the indicated genotypes under MS5-DL4 or MS5 co-culture conditions (mean ± SD, n=3 independent experiments).two-way ANOVA; p-value ****<0.0001,

c) Cell death analysis of *LMO1*^{tg} pre-LSCs co-cultured on MS5-DL4 stromal cells following drug treatment (% of early apoptotic, late apoptotic and necrotic cells at 24-72 h) (mean ± SD, n=3 independent experiments). Two-way ANOVA, p-values *=0.045; **=0.0013; ***=0.0002; and ****<0.0001,

d) Cell death analysis of *SCL*^{tg}*LMO1*^{tg} pre-LSCs co-cultured on MS5-DL4 stromal cells following drug treatment (% of early apoptotic, late apoptotic and necrotic cells at 24-72 h) (mean ± SD, n=3 independent experiments).

e) Viability analysis of MS5-DL4 stromal cells following drug treatment. MS5-DL4 stromal cells were grown in presence of different concentrations of IACS-010759. Shown is the percentage of living MS5-DL4 stromal cells.

f) Representative DIC image of cell cultures after 48h treatment with the indicated dose of IACS-010759, scale bar 20 uM, from n=3 independent experiments .

Supplementary Figure 3

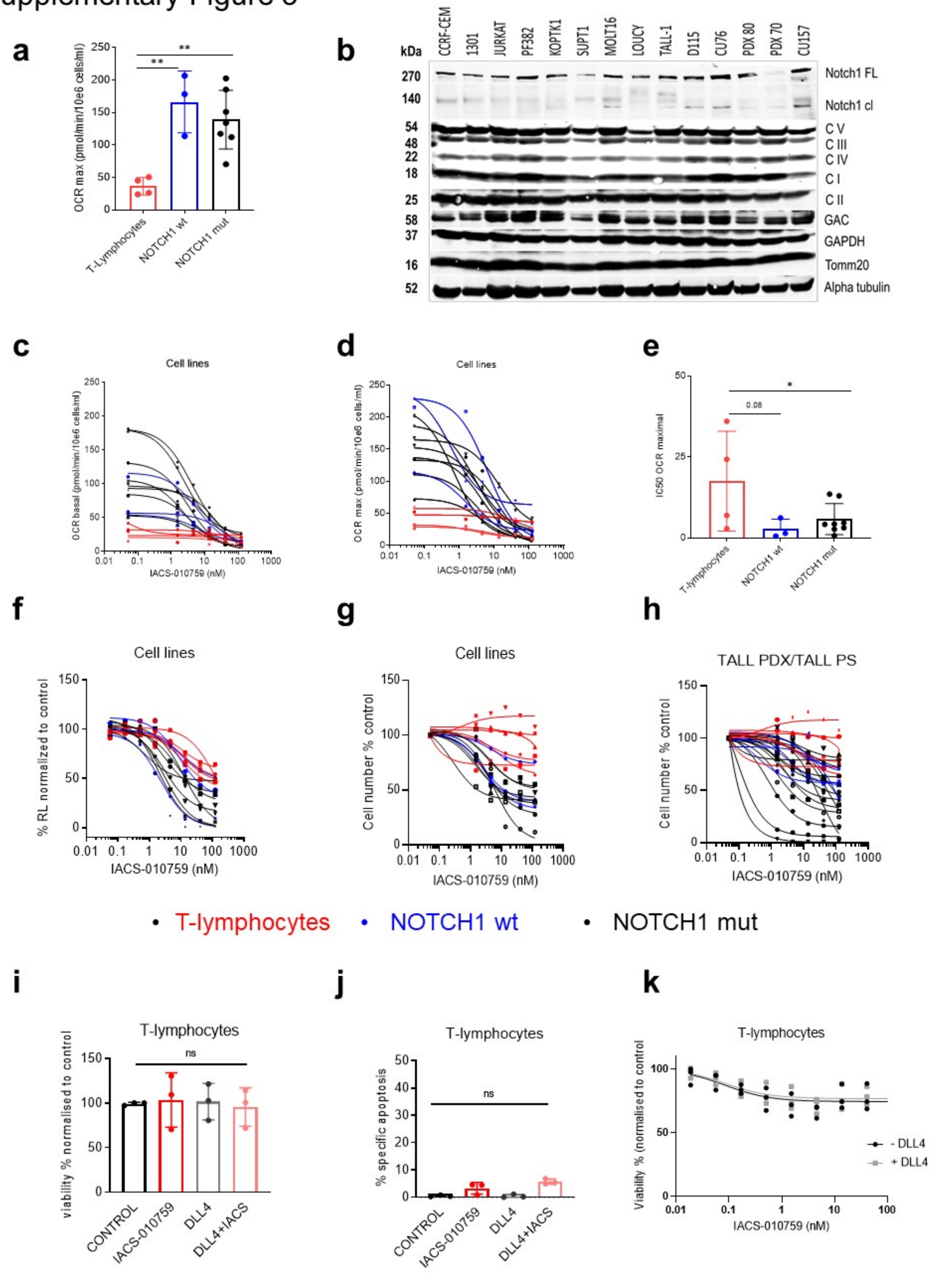

Supplementary Figure 3. NOTCH1 mutations sensitizes leukemic cells to OxPhos inhibition.

a) Maximal oxygen consumption rates (OCR) in *NOTCH1*-mutated (black) (n=7) and *NOTCH1* wild type (-wt) (blue) T-ALL cell lines (n=3) and T-lymphocytes (red) (n=4) by Mito Stress Test assay (Seahorse Technology) (mean±SD, n=3 independent experiments for each cell line with n=4 technical replicates/condition);.one-way ANOVA; p-value **=0.0014; ***=0.0002;

b) Western blot of electron transport chain complexes I-V, glutaminase (GAC), GAPDH, α-tubulin, and Tomm20 in panel of *NOTCH1*-mutated and *NOTCH1*-wt T-ALL cell lines and PDX samples, representative of at least 2 experimental replicates.

c) Basal OCR (Mito Stress Test assay, Seahorse Technology) after 4 hr treatment with 0-123 nM of IACS-010759, for *NOTCH1*-mutated (black)(n=7) and *NOTCH1*-wt (blue) (n=3) T-ALL cell lines and healthy T-lymphocytes (red) (n=4), (mean±SD, n=3 independent experiments per cell line with n=4 technical replicates/condition).

d) Maximal OCR (Mito Stress Test assay) after 4 h treatment with 0-123 nM of IACS-010759, for each *NOTCH1*-mutated (black)(n=7) and *NOTCH1*-wt (blue)(n=3) T-ALL cell lines and healthy T-lymphocytes (red);(mean±SD was calculated as a summary of means for each cell line/t-lymphocyte sample of n=4 technical replicates);

e) IC_{50} values for maximal OCR inhibition in (d) for *NOTCH1*-mutated (black) and *NOTCH1*-wt (blue) T-ALL cell lines and healthy T-lymphocytes (red); (mean±SD; n=3 independent experiments with n=4 technical replicates/condition); one-way ANOVA; p-value *=0.02; **=0.004;

f) Viability analysis of *NOTCH1*-wild type (blue) and *NOTCH1*-mutated (black) T-ALL cell lines and healthy T-lymphocytes (red), treated with 0-123 nM of IACS-010759 for 96 hrs (CTG assay) (mean±SD was calculated as a summary of means for each cell line type/t-lymphocyte sample of n=4 technical replicates, from 3 independent experiments).

g) Viability analysis of *NOTCH1*-wild type (blue) and *NOTCH1*-mutated (black) T-ALL cell lines and healthy T-lymphocytes (red), treated with 0-123 nM of IACS-010759 for 96 hrs (flow cytometry) (mean±SD; n=3 independent experiments per line with n=3 technical replicates).

h) Viability analysis of *NOTCH1*-wild type (blue) and *NOTCH1*-mutated (black) T-ALL patient samples and PDX models and healthy T-lymphocytes (red), treated with 0-123 nM of IACS-010759 for 96 hrs (flow cytometry).

i) Viability analysis of healthy T-lymphocytes treated in presence or absence of DLL4 and subjected to treatment with 10 nM of IACS-010759 or DMSO for 96 hrs (CTG assay) (mean±SD, n=3 independent experiments, with n=3 technical replicates/condition); ns - calculated using one sided ANOVA test.

j) Viability analysis of healthy T-lymphocytes, treated in presence or absence of DLL4, treated with 10 nM of IACS-010759 or DMSO for 96 hrs (flow cytometry)(mean±SD, n=3 independent experiments with n=3 replicates/condition); ns -calculated using one sided ANOVA test.

k) Viability analysis of healthy T-lymphocytes, in presence or absence of DLL4 , treated with 0-123 nM of IACS-010759 for 96 hrs (CTG assay) (mean±SD, n=3 independent experiments with n=3 replicates).

Supplementary Figure 4

a Mito stress test for IACS-010759 in *NOTCH1* wild type cell lines

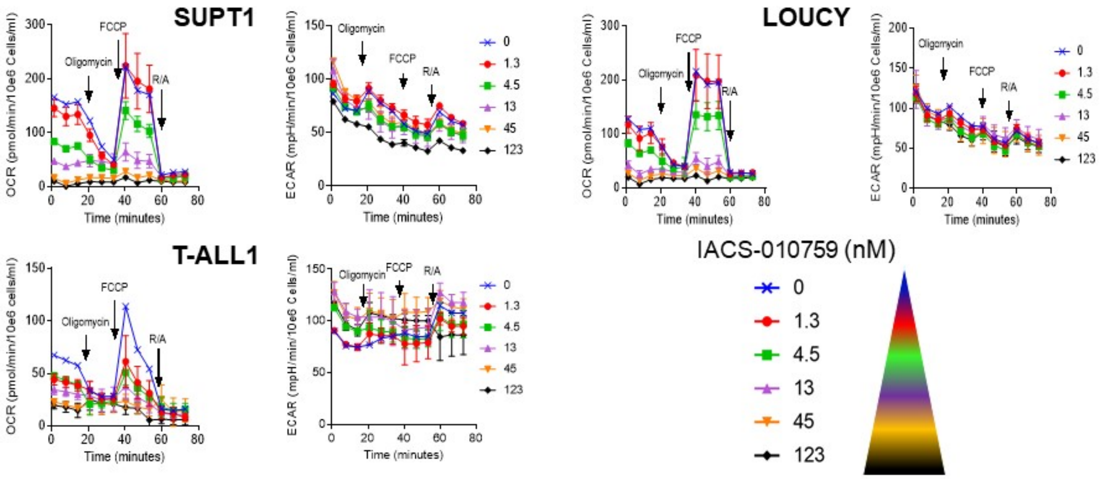

b Mito stress test for IACS-010759 in *NOTCH1* mutant cell lines

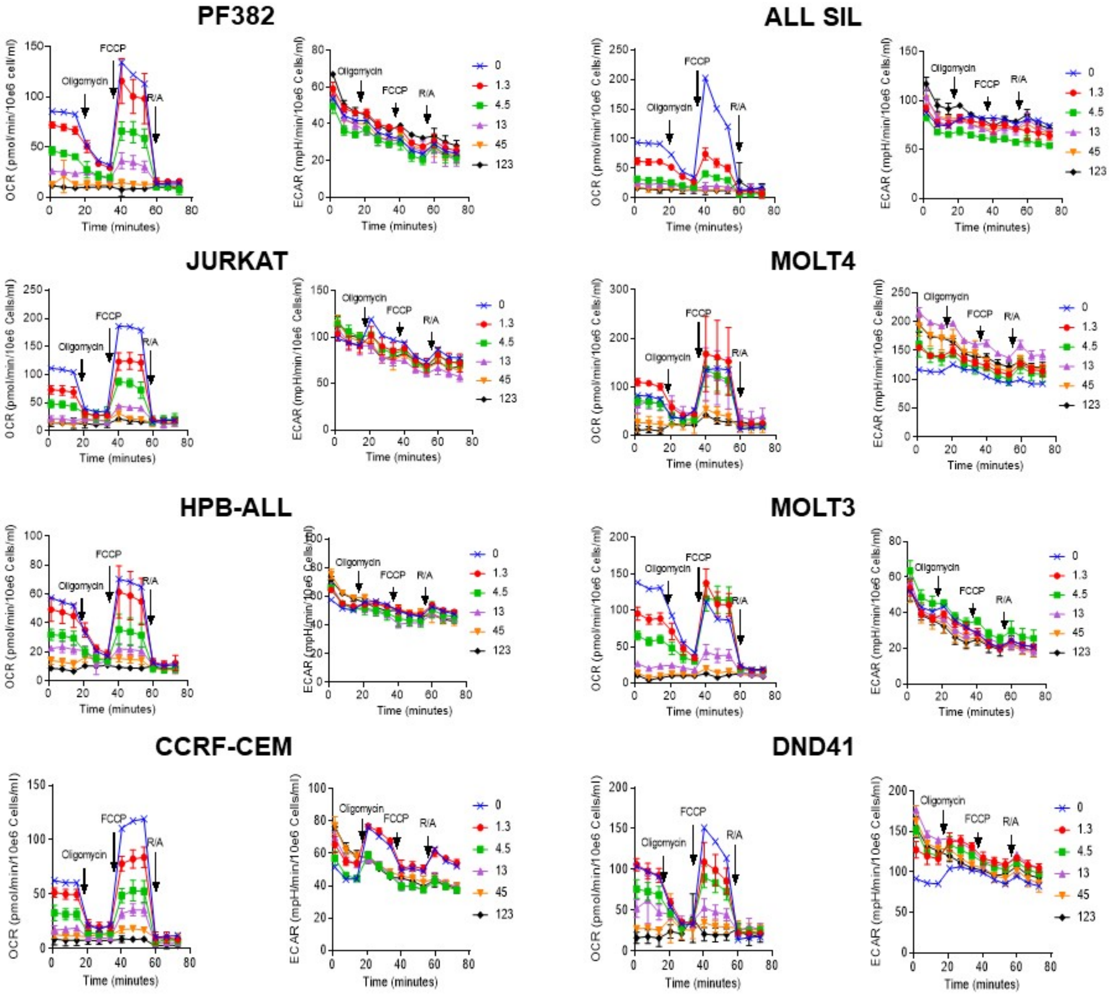

Supplementary Figure 4. IACS-010759 inhibits Oxygen consumption rate in NOTCH1 wild type and mutant T-ALL cell lines

a) Representative graphs of Oxygen consumption rate (OCR) (left) and extracellular acidification rate (ECAR) (right) response during Mito Stress Test in NOTCH1-wt T-ALL cell lines after 4 h treatment with the indicated concentrations of IACS-010759 (mean±SD, n=3 independent experiments, n=4 replicates/condition).

b) Representative OCR (left) and ECAR (right) during Mito Stress Test in NOTCH1-mutated T-ALL cell lines after 4 hr treatment with the indicated concentrations of IACS-010759 (mean±SD, n=3 independent experiments, n=4 replicates/condition).

Supplementary Figure 5

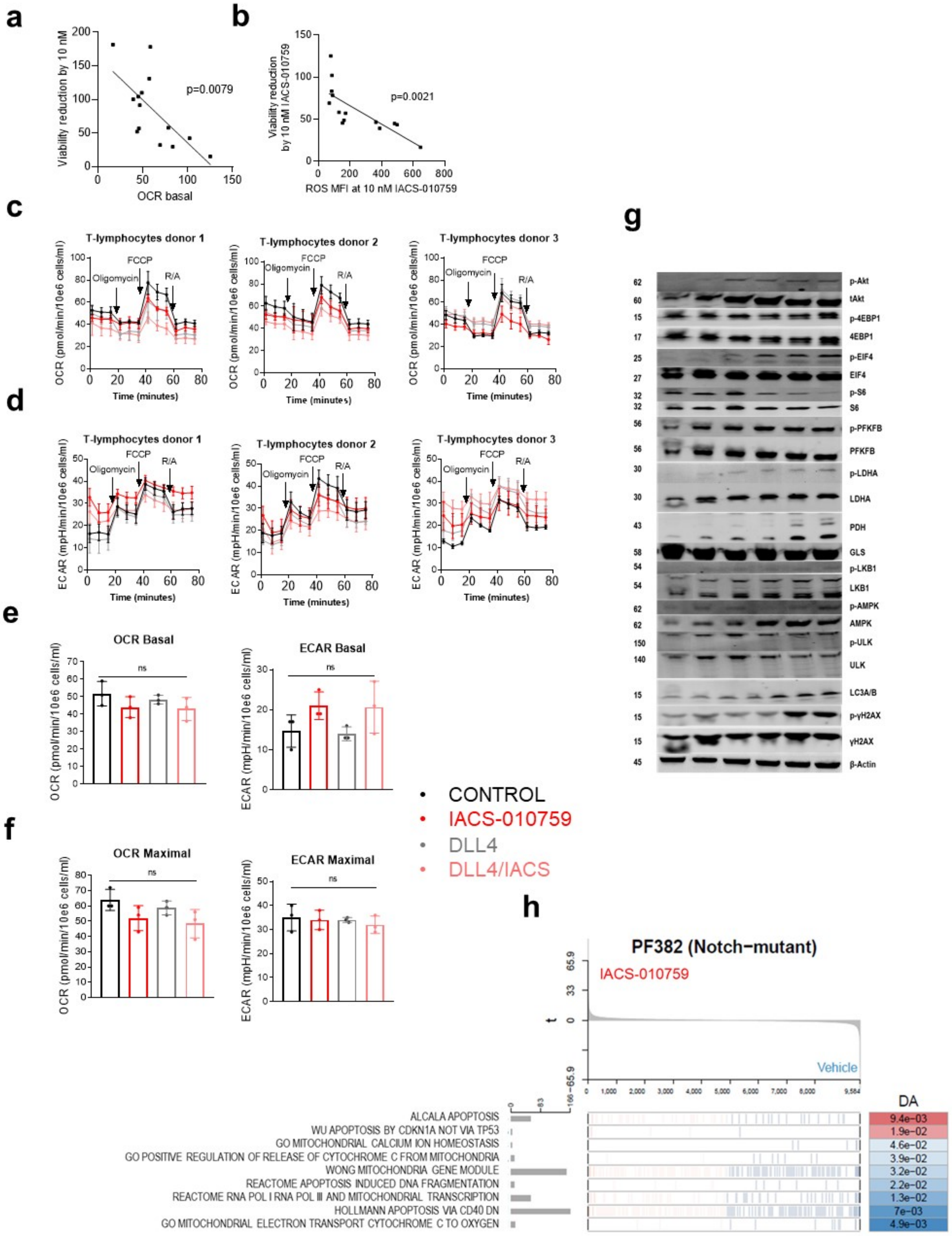

Supplementary Figure 5. DLL4 does not impact response to OxPhos-i in T-lymphocytes

a) Correlation of viability reduction and decrease in OCR in T-ALL cell lines and T-lymphocytes exposed to IACS-010759 at 10 nM; simple liner regression: F=10.11; DFn,DFd=1,12; P value=0.0079; R squared =0.4572

b) Correlation analysis of cell viability reduction after treatment with IACS-010759 and increase in ROS across T-ALL cell lines and T-lymphocytes; (mean±SD, n=3 independent experiment); simple linear regression F=15.21; DFn,DFd=1,12; P-value=0.0021; R squared =0.5590;

c) Oxygen consumption rate (OCR) measured on healthy T-lymphocytes following culture with or without DLL4, treated with DMSO or 10 nM of IACS-010759; Mito Stress Test assay, by Seahorse;(mean±SD, n=4 replicates/condition)

d) Extracellular acidification rate (ECAR) measured on healthy T-lymphocytes following culture with or without DLL4, treated with DMSO or 10 nM of IACS-010759; Mito Stress Test assay, by Seahorse; (mean±SD, n=4 replicates/condition)

e) Basal OCR and basal ECAR from the experiment described in (C) and (D) (mean±SD, n=3 independent donors, n=4 replicates/condition), ns-no significance; one-way ANOVA;

f) Maximal OCR and maximal ECAR from the experiment described in (C) and (D) (mean±SD, n=3 independent donors, n=4 replicates/condition), ns-no significance; one-way ANOVA;

g) Representative Western blots of protein expression changes over 24 h after treatment with 10 nM IACS-010759 in the *NOTCH1*-mutated cell line JURKAT. Primary antibodies against proteins involved in the mTOR/Akt pathway, AMPK pathway, glycolysis pathway, DNA damage response, and autophagy were used, results representative of n=3 experimental replicates.

h) Top: KEGG signaling pathways affected by IACS-019759 in NOTCH1-mutated cell line PF-382; Bottom: Gene Ontology analysis of genes affected by IACS-010759 treatment.

Supplementary Figure 6

a Nutrient dependency in examples of *NOTCH1* mutant cell lines

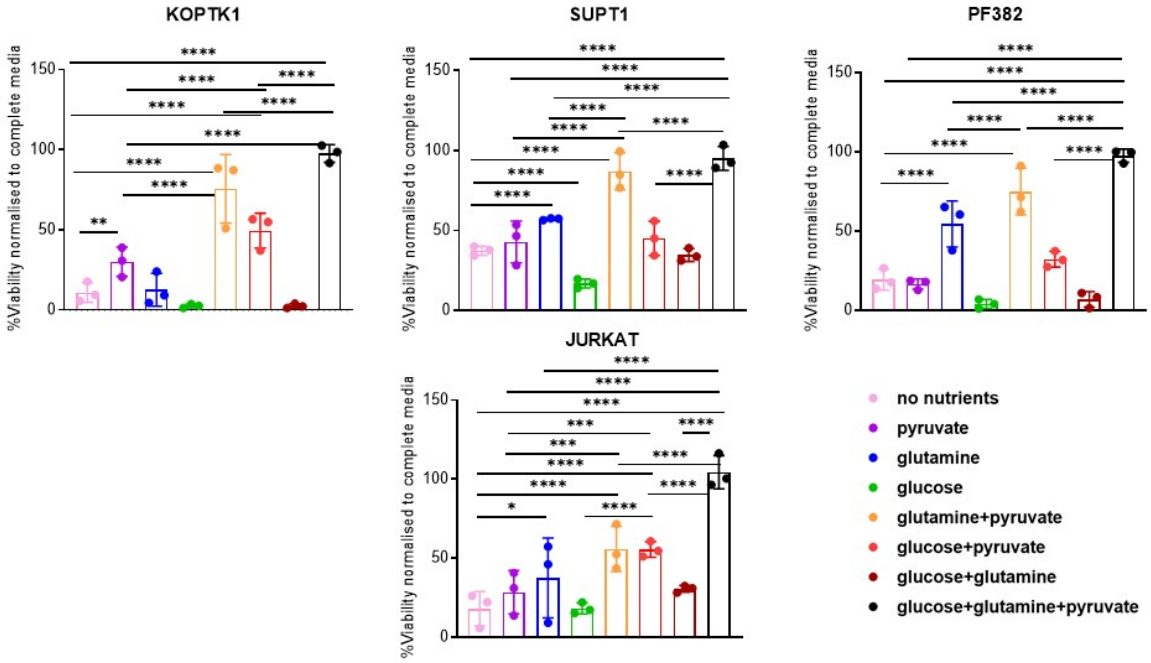

b ECAR, OCR basal and max upon nutrient withdrawal in *NOTCH1* mutant cell lines

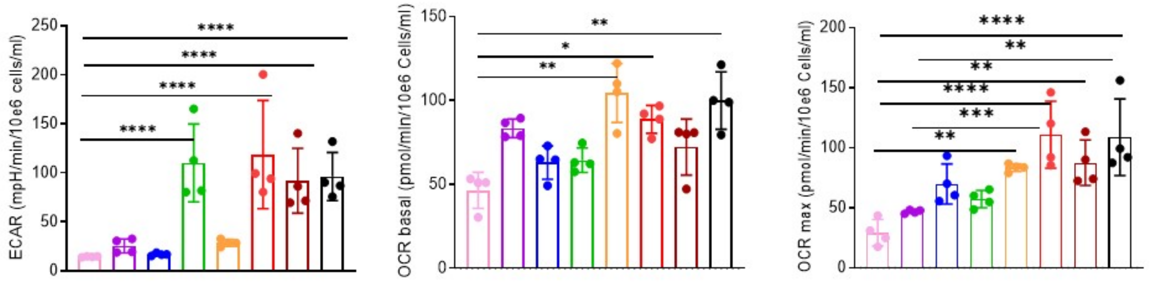

c Nutrient dependency in *NOTCH1* wild type cell lines

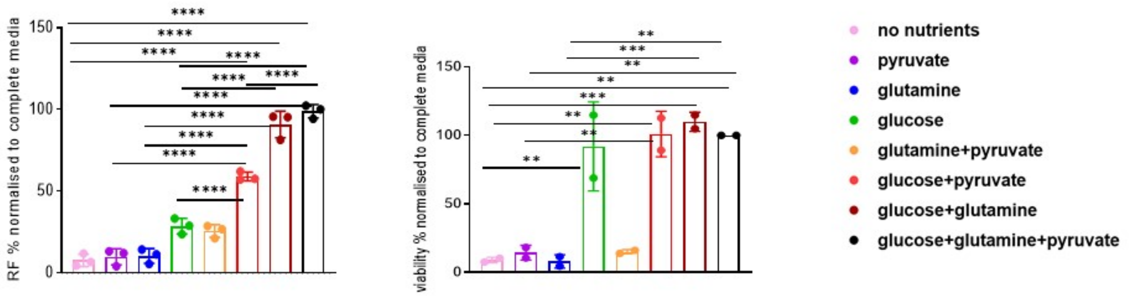

Supplementary Figure 6. Selective nutrient starvation uncovers different metabolic dependencies in T-ALL cell lines

a) Analysis of cell growth for KOPT-K1, SUP-T1, JURKAT, and PF-382 T-ALL cell lines in the context of nutrient starvation by culture in media containing: no nutrients, pyruvate, glutamine, glucose, glutamine + pyruvate, glucose + pyruvate, glucose + glutamine, or glucose + pyruvate + glutamine, measured by CTG assay (mean±SD, n=3 independent experiments, in triplicates);

b) Summary of the results for the 4 NOTCH1-mutated T-ALL cell lines showing maximal OCR in nutrient-starvation conditions (mean±SD, n=3 independent experiments, with n= 4 replicates);

c) Analysis of cell growth in T-ALL NOTCH1-wt cell lines in the context of nutrient starvation by culture cells in media containing: no nutrients, pyruvate, glutamine, glucose, glutamine + pyruvate, glucose + pyruvate, glucose + glutamine, or glucose + pyruvate + glutamine, measured by CTG assay (mean±SD n=3 independent experiments, in triplicates) and flow cytometry (mean±SD n=2 independent experiments, in triplicates);

One-way ANOVA: P-values: *<0.05; **<0.005; ***<0.001; and ****<0.0001.

Supplementary Figure 7

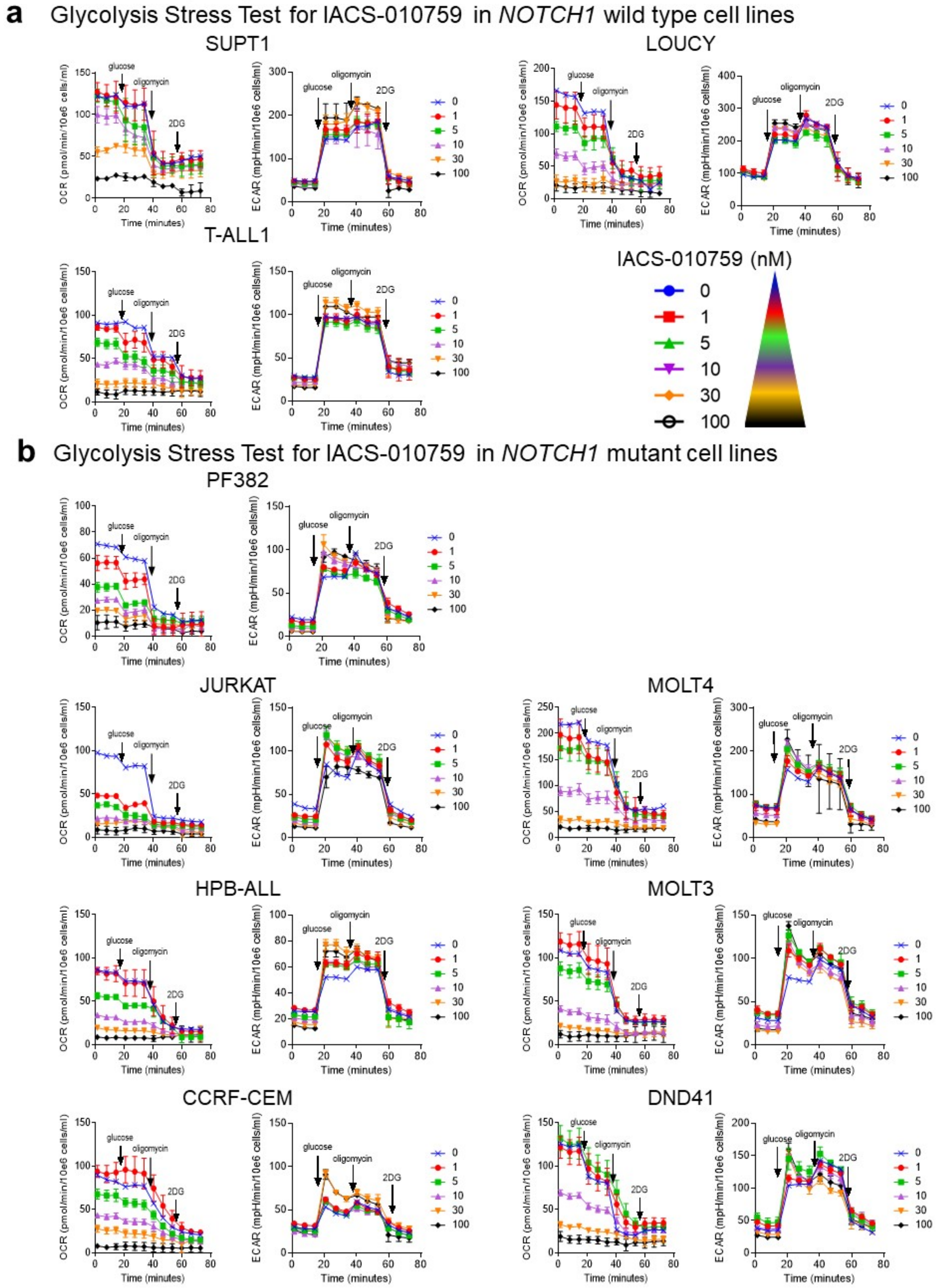

Supplementary Figure 7. NOTCH1 controls glycolytic capacity upon OxPhos blockade

a) Representative graphs of Oxygen consumption rate (OCR) (left) and extracellular acidification rate (ECAR) (right) during Glycolysis Stress Test in NOTCH1-wt T-ALL cell lines after 4 hr treatment with the indicated concentrations of IACS-010759 (mean±SD, n=3 independent experiments, n=4 replicates/condition).

b) Representative graphs of OCR (left) and ECAR (right) during Glycolysis Stress Test in NOTCH1-mutated T-ALL cell lines after 4 hr treatment with the indicated concentrations of IACS-010759 (mean±SD, n=3 independent experiments with n=4 replicates/condition).

Supplementary Figure 8

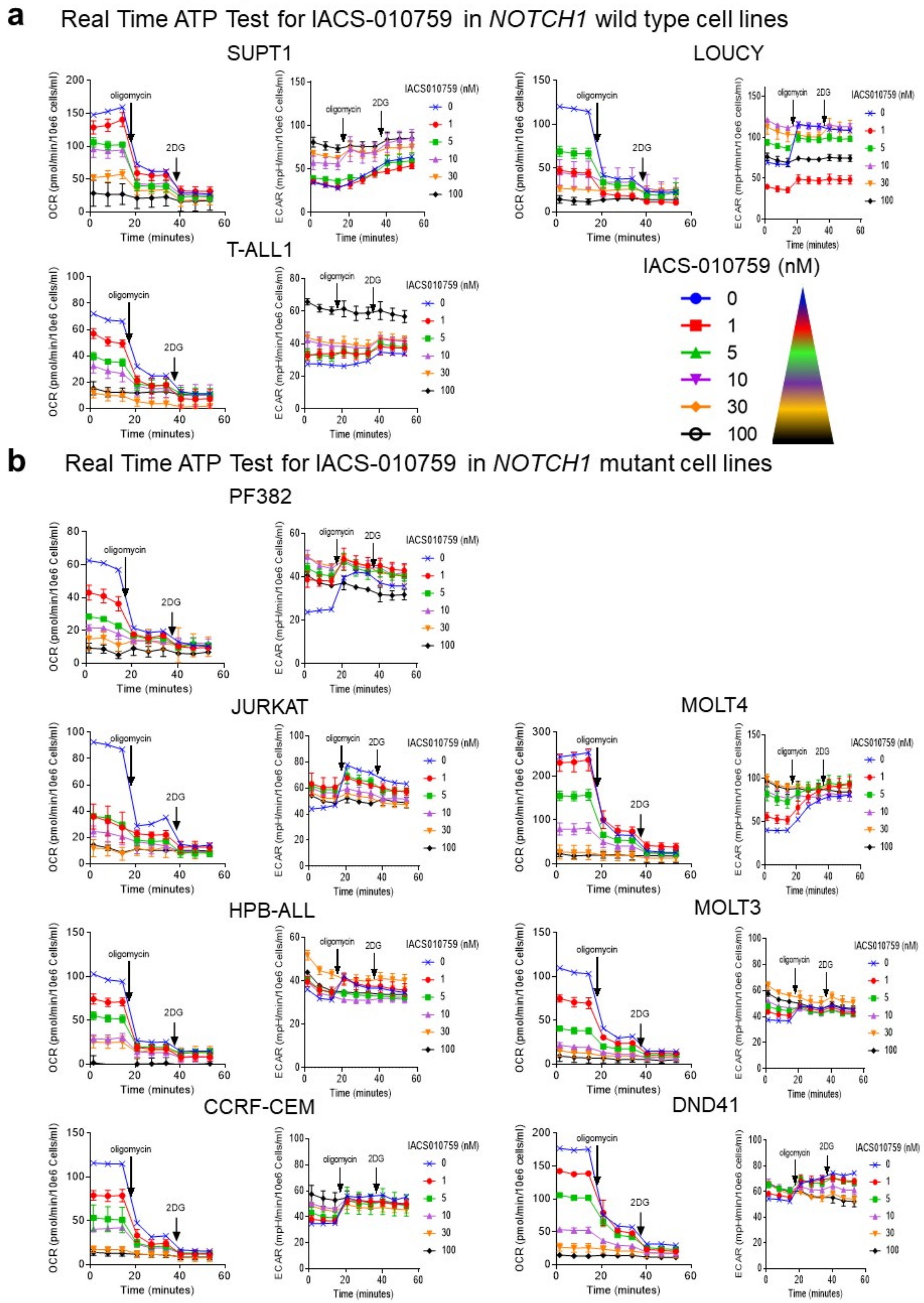

Supplementary Figure 8. NOTCH1 status impacts ATP generation upon OxPhos blockade

a) Representative graphs of OCR (left) and ECAR (right) during Real Time ATP Assay in NOTCH1-wt T-ALL cell lines after 4 hr treatment with the indicated concentrations of IACS-010759 (mean±SD, n=3 independent experiments, n=4 replicates/condition).

b) OCR (left) and ECAR (right) during Real Time ATP Assay in NOTCH1-mutated T-ALL cell lines after 4 hr treatment with the indicated concentrations of IACS-010759 (mean±SD, n=3 independent experiments, with n=4 replicates/condition).

Supplementary Figure 9

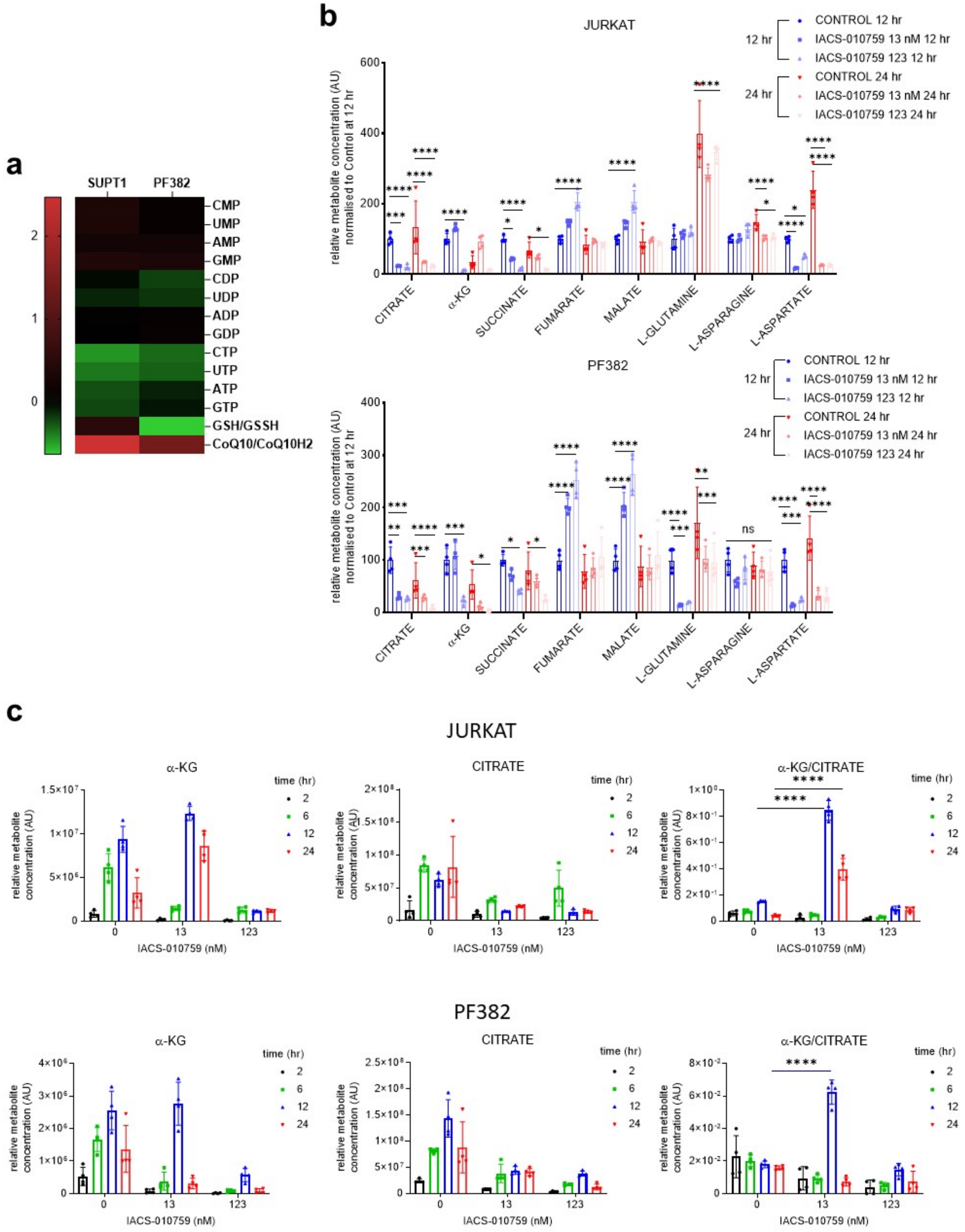

Supplementary Figure 9. IACS-010759 inhibits TCA cycle in time- and dose-dependent manner

a) Modulation of selected intracellular metabolites in NOTCH1-mutated T-ALL cell line PF-382 and NOTCH1-wild type cell line SUP-T1 after treatment with IACS-010759 or DMSO for 24 hrs. Levels shown are expressed as log2 of the ratio of metabolite levels in cells treated with IACS-010759 to metabolite levels in cells treated with DMSO;

b) Intracellular modulation of selected metabolites following treatment with IACS-010759 in the NOTCH1-mutated cell lines JURKAT and PF-382. Cells were treated for 12 and 24 hrs with 0 nM, 13 nM, or 123 nM IACS-010759. Intracellular metabolite extracts were analyzed using ultra-performance liquid chromatography-tandem mass spectrometry. Concentrations were normalized to the average metabolite level in the control samples (treated with 0 nM IACS-010759) at 12 h. Mean ± SD (4 replicates) relative concentrations are shown. Significance was determined using multiple comparisons two-way ANOVA; ns-no significant, p-values are shown * < 0.05; ** < 0.01; *** = 0.0001; **** < 0.0001;

c) Mass spectrometry measured α-ketoglutarate and citrate levels and the ratio of α-ketoglutarate/citrate, at 2, 4, 6 and 12 hr and upon treatment with DMSO, 13 or 123 nM of IACS-010759 (mean±SD, n=1 with 4 technical replicates, for two cell lines JURKAT and PF382). Significance was determined using a one-sided Student t-test (to compare either 13 or 123 nM IACS-010759 versus control) followed by the false discovery rate (FDR). The changes over time were compared using two-way ANOVA, p-values are shown as: * < 0.05; ** < 0.01; *** < 0.001; **** < 0.0001;

Supplementary Figure 10

Glucose labeling for IACS-010759 in *NOTCH1* mutant

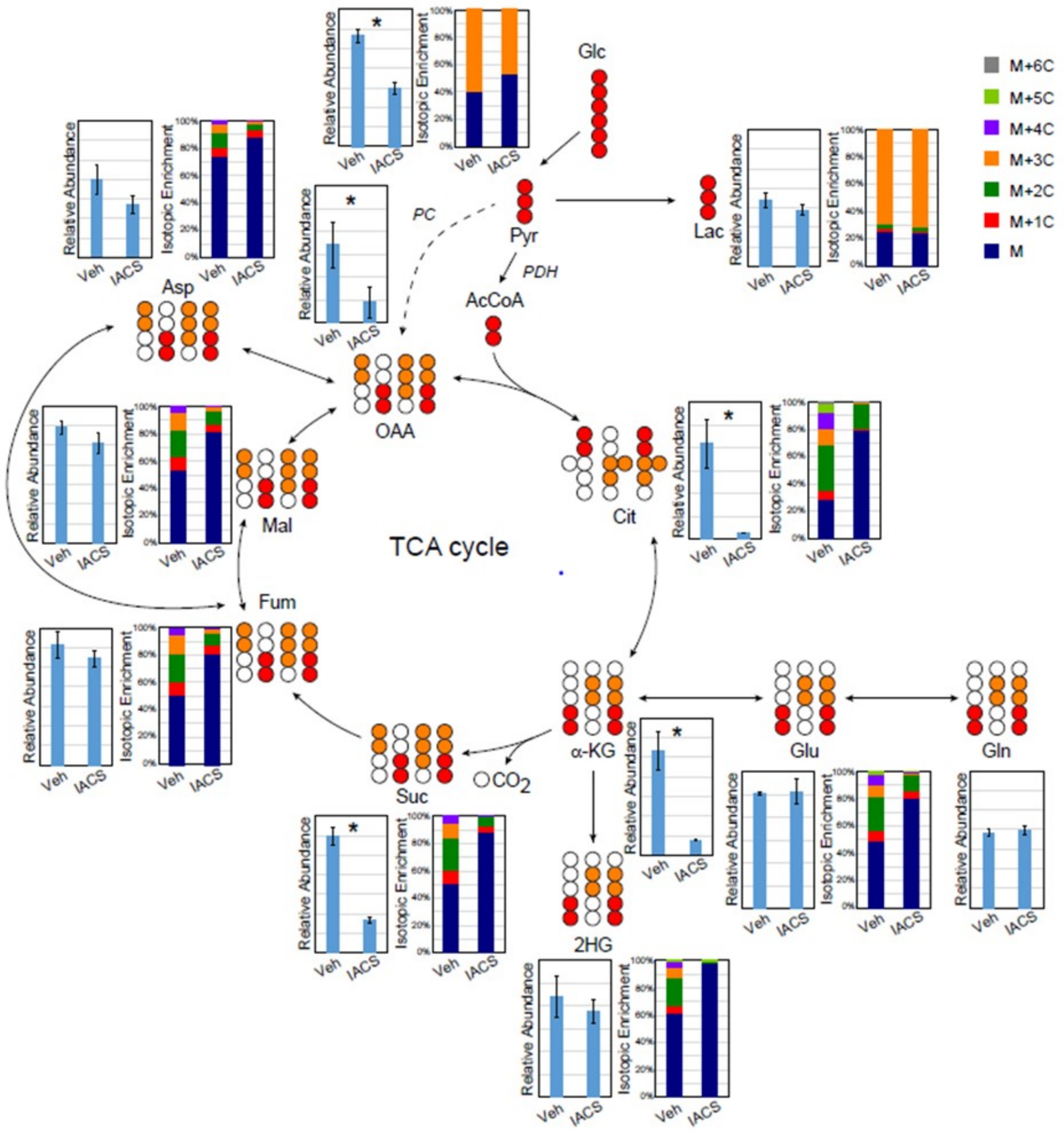

Supplementary Figure 10. Glucose labelled flux analysis shows changes in TCA cycle activity upon OxPhos blockade in NOTCH1 mutant cell line PF-382

Effects of IACS-010759 at a dose of 10 nM after 12 hrs on the metabolic fluxes in NOTCH1-mutated T-ALL cell line PF-382 cultured with $^{13}C_6$ glucose were analyzed using stable isotope-resolved metabolomics and ultra-performance liquid chromatography-tandem mass spectrometry.

Supplementary Figure 11

a Glucose labeling for IACS-010759 in *NOTCH1* wild type

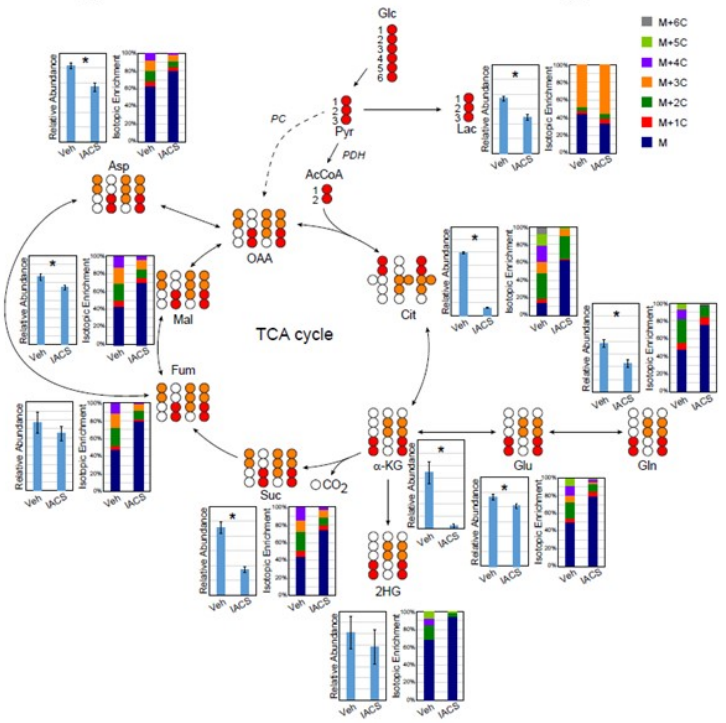

b Glutamine labeling for IACS-010759 in *NOTCH1* wild type

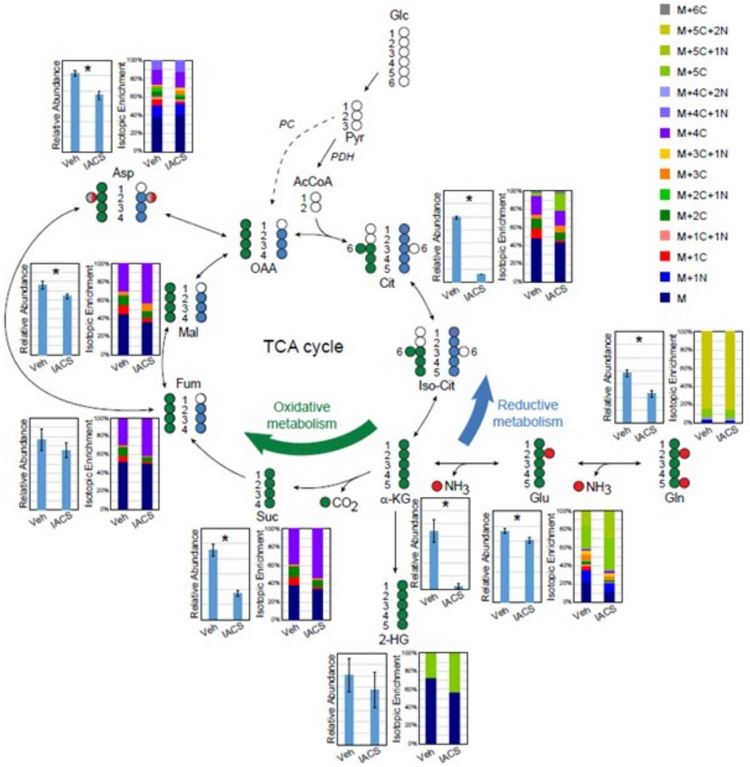

Supplementary Figure 11. Glucose and Glutamine labelled flux analysis shows changes in TCA cycle activity and utilization of reductive metabolism of glutamine upon OxPhos blockade in NOTCH1 wild type cell line SUP-T1

Effects of IACS-010759 at a dose of 10 nM on the metabolic fluxes in *NOTCH1*-wild type T-ALL cell line SUP-T1 cultured with (A) $^{13}C_6$ glucose or (B) $^{13}C_5$, $^{15}N_2$-glutamine were analyzed using stable isotope-resolved metabolomics and ultra-performance liquid chromatography-tandem mass spectrometry.

Supplementary Figure 12

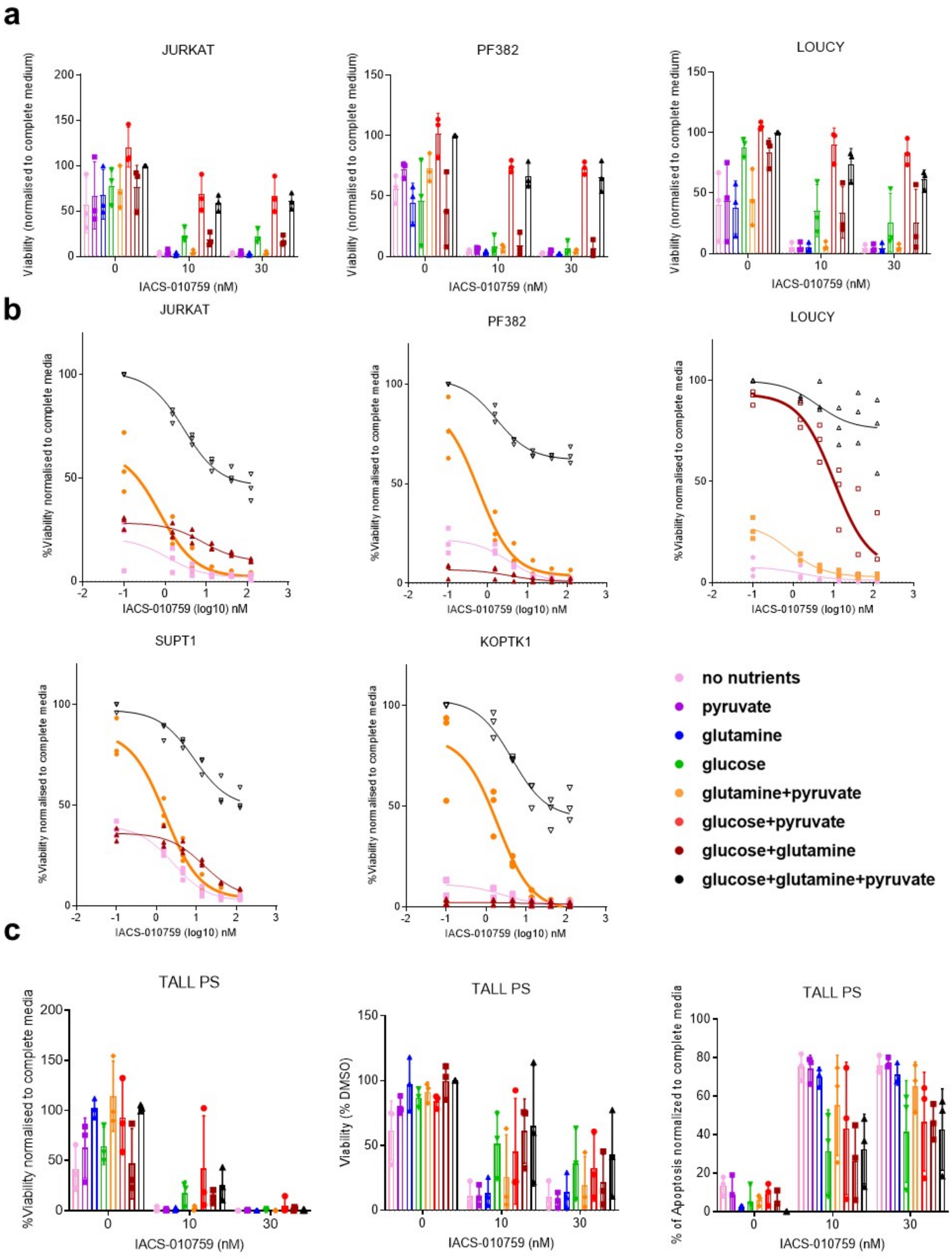

Supplementary Figure 12. Selective depletion of nutrients modulates response to OxPhos inhibition

a) Viability of NOTCH1-mutant T-ALL cell lines JURKAT and PF-382 and NOTCH1-wt T-ALL cell line LOUCY under treatment with the indicated concentrations of IACS-01759 (0-100 nM) under the following nutrient conditions: no nutrients, pyruvate (Pyr), glutamine (Gln), glucose (Glc), glutamine + pyruvate, glucose + pyruvate, glucose + glutamine, glucose + pyruvate + glutamine (mean±SD, n=3 independent experiments, with n=3 replicates per condition)

b) Viability of *NOTCH1*-mutated T-ALL cell lines JURKAT, PF-382, and KOPT-K1 and *NOTCH1*-wild type T-ALL cell lines LOUCY and SUP-T1 treated with the indicated concentrations of IACS-01759 (0-100 nM) under the following nutrient conditions: no nutrients, glutamine + pyruvate, glucose + pyruvate + glutamine, as measured by CTG assay (mean±SD, n=3 independent experiments, with n=3 replicates per condition)

c) Analysis of response to IACS-010759 and nutrient deprivation in T-ALL patient samples (PS). From left to right: CTG assay showing cell viability, flow cytometry showing viable cells, Annexin V flow cytometry showing apoptotic cells (mean±SD, n=3 independent patient samples, with n=3 replicates per condition)

Supplementary Figure 13

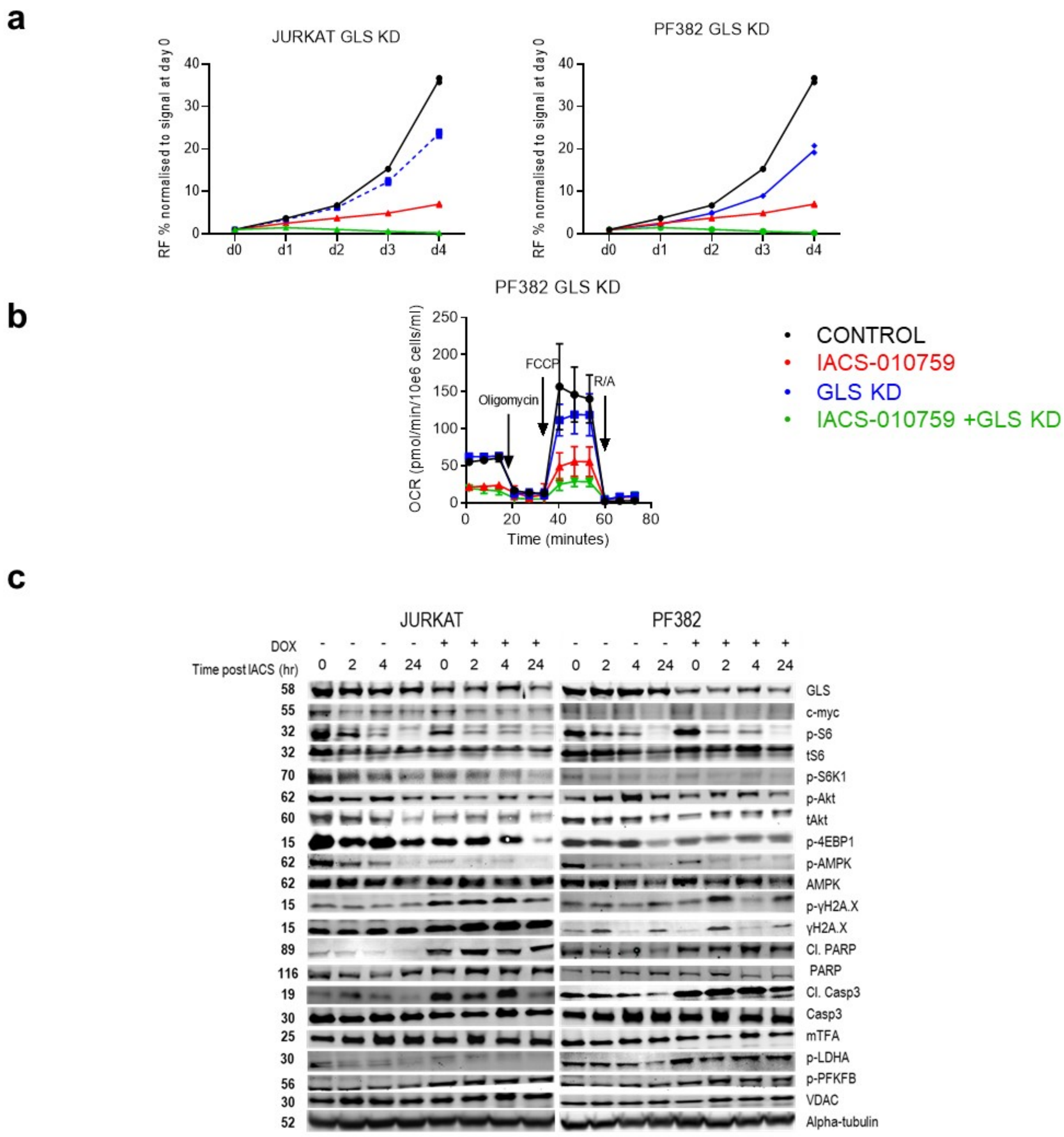

Supplementary Figure 13. OxPhos blockade and Glutaminase knockdown contribute to blockade of oxidative respiration and cell growth in NOTCH1 mutated cell lines JURKAT and PF382

a) Time course of cell viability under treatment with 10 nM IACS-010759 (red), induction of GLS knockdown with doxycycline at 2 μg/mL (blue), or the combination of both (green) in JURKAT (left) and PF-382 (right) T-ALL cell lines, normalized to the initial signal at day 0, as measured by CTG assay (mean±SD, n=3 independent experiments, with n=3 replicates per condition).

b) Representative graph of OCR determined by Mito Stress Test assay in the NOTCH1-mutated T-ALL cell lines PF-382 subjected to doxycycline-induced knockdown of GLS (blue), treated with IACS-010759 (10 nM) for 4 h (red), or the combination of both (green) (mean±SD, n=3 independent experiments, with n=4 replicates per condition).

c) Time-course immunoblotting analysis of *NOTCH1*-mutated cell lines JURKAT and PF-382 with or without doxycycline-induced knockdown of GLS, after treatment with 10 nM IACS-010759. All immunoblotting data are representative of n=3 independent experiments.

Supplementary Figure 14

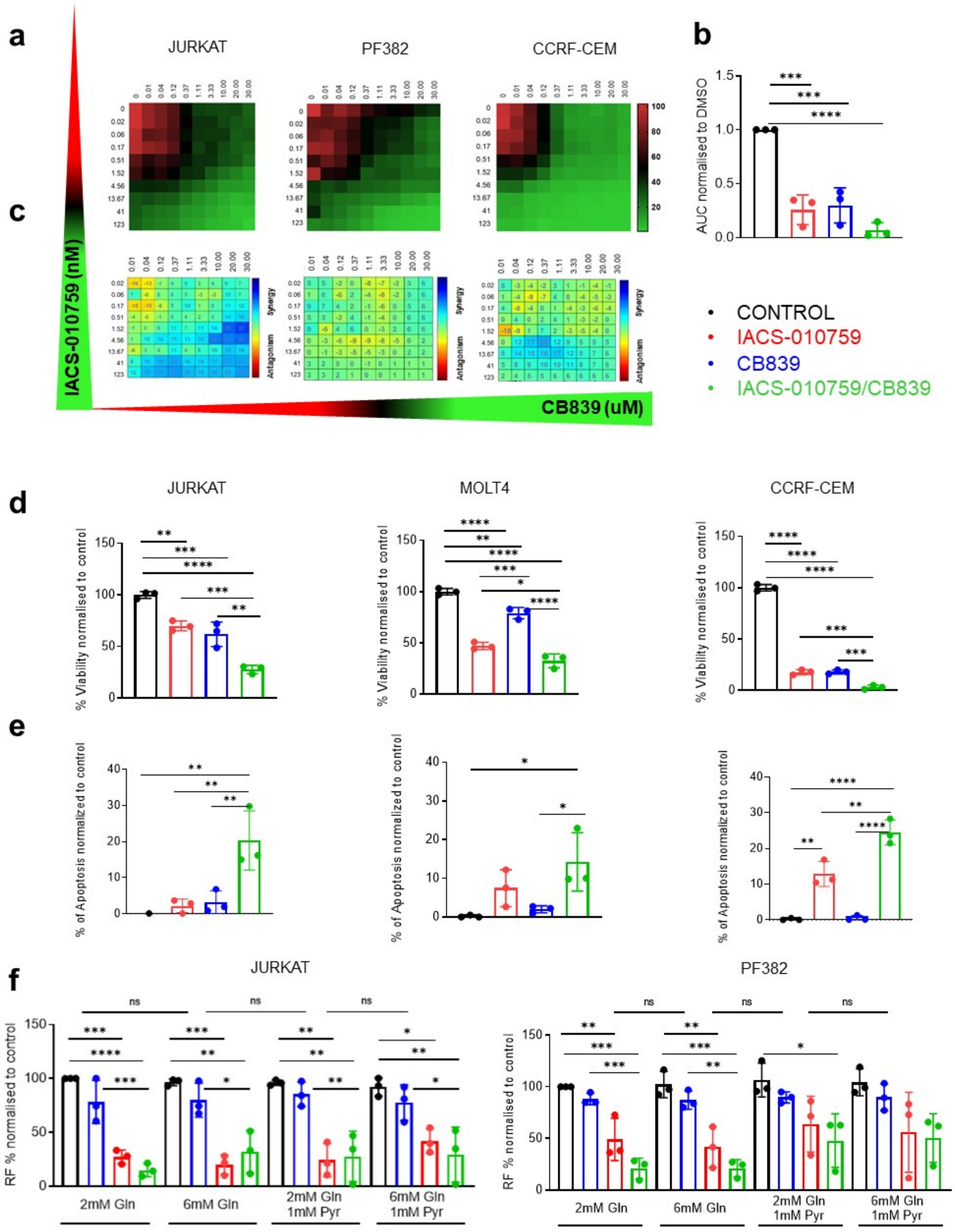

Supplementary Figure 14. Dual OxPhos and Glutaminase blockade causes synergistic inhibition of cell viability and induces apoptosis in NOTCH1 mutated cell lines

a) Matrix of cell viability under treatment with the indicated doses of CB-839 (0-30 µM) and IACS-01759 (0-123 nM) in T-ALL cell lines JURKAT, PF-382, and CCRF-CEM. Each square of the concentration matrix represents a mean value, normalized to levels in DMSO-treated controls, from 5 independent experiments, as measured by CTG assay

b) Area under the curve (AUC) of cell viability in T-ALL cell lines (JURKAT, PF-382, and CCRF-CEM) treated with increasing doses of CB-839 (0-30 µM) and IACS-01759 (0-123 nM) (mean±SD, n=3 cell lines, with n=3 independent experiments and n=3 replicates per condition);one-way ANOVA; p-value ***<0.001

c) Results of delta BLISS index calculations for T-ALL cell lines using COMBENEFIT software provided by Cancer Research UK Cambridge Institute.

d) Summary of analysis of cell viability after 96 h treatment with 1 µM CB-839 and 10 nM IACS-010759 in *NOTCH1*-dependent T-ALL cell lines JURKAT, MOLT-4, and CCRF-CEM. (mean±SD, n=3 independent experiments with n=3 replicates per condition); one-way ANOVA: p-value *=0.02; **=0.002; ***=0.0002; and ****<0.0001,

e) Summary of analysis of cell apoptosis after 96 h treatment with 1 µM CB-839 and 10 nM IACS-010759 in *NOTCH1*-dependent T-ALL cell lines JURKAT, MOLT-4, and CCRF-CEM. (mean±SD, n= 3 independent experiments with n=3 replicates per condition) measured by flow cytometry; one-way ANOVA: p-value *=0.02; **=0.0011; ****<0.0001,

f) Cell viability rescue experiment under treatment with 1 µM CB-839 and 10 nM IACS-010759 in *NOTCH1*-dependent T-ALL cell lines JURKAT and PF-382 for 96 hr, with or without exposure to 2 mM glutamine (Gln); 6 mM glutamine; 2 mM glutamine + 1 mM pyruvate (Pyr); or 6 mM glutamine + 1 mM pyruvate. (mean±SD, n= 3 independent experiments with n=3 replicates per condition), measured by CTG assay. Two-way ANOVA; p-values: ns-no significant, *=0.01; **=0.0013; ***=0.0003; and ****<0.0001.

Supplementary Figure 15

a Mito stress test for CB839 and IACS-010759 in *NOTCH1* wild type cell lines

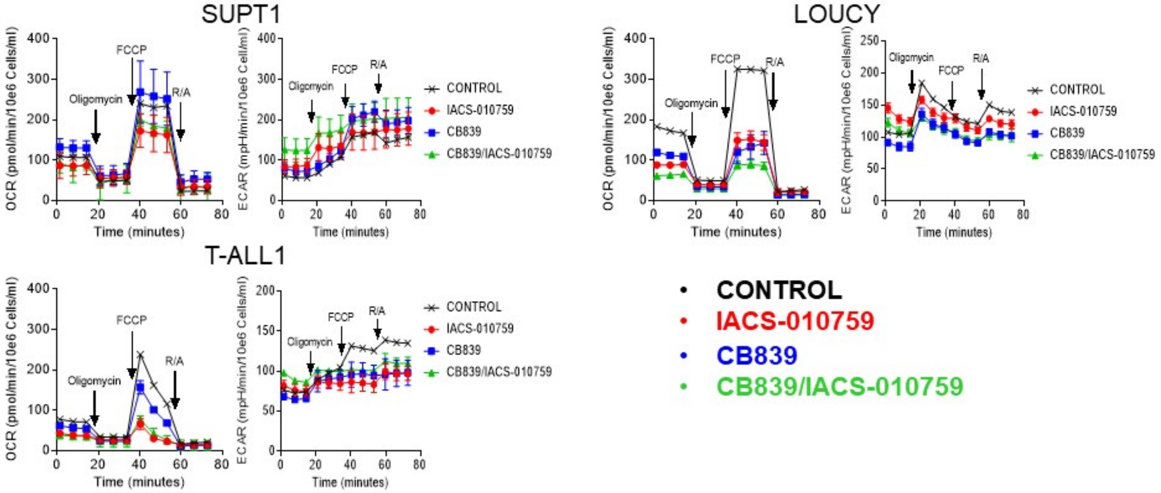

b Miro stress test for CB839 and IACS-010759 in *NOTCH1* mutated cell lines

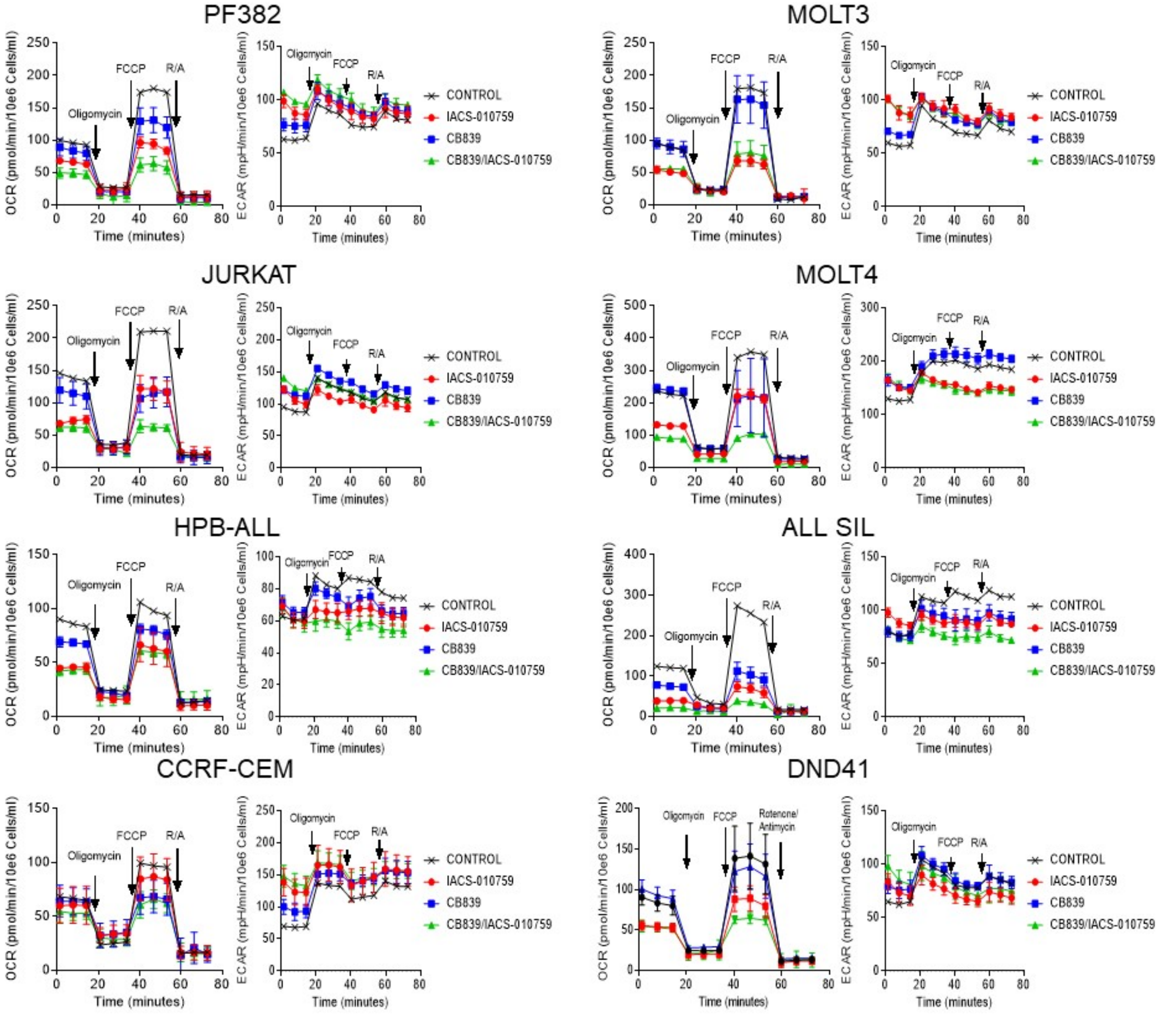

Supplementary Figure 15. Dual blockade of OxPhos and Glutaminase blocks oxidative respiration in NOTCH1 wild type and NOTCH1 mutant cell lines

a) Representative graphs of OCR (left) and ECAR (right) determined by Mito Stress Test in NOTCH1-wt T-ALL cell lines after 4 hr treatment with vehicle (control), 10 nM of IACS-010759, 12 hr treatment with 1 μM of CB-839;(mean±SD, n=3 independent experiments with n=4 replicates per condition);

b) Representative graphs of OCR (left) and ECAR (right) during Mito Stress Test in NOTCH1-mutated T-ALL cell lines after 4 hr treatment with 10 nM of IACS-010759, 12 hr treatment with 1 μM of CB-839, or the combination of both; (mean±SD, n=3 independent experiments with n=4 replicates per condition);

Supplementary Figure 16

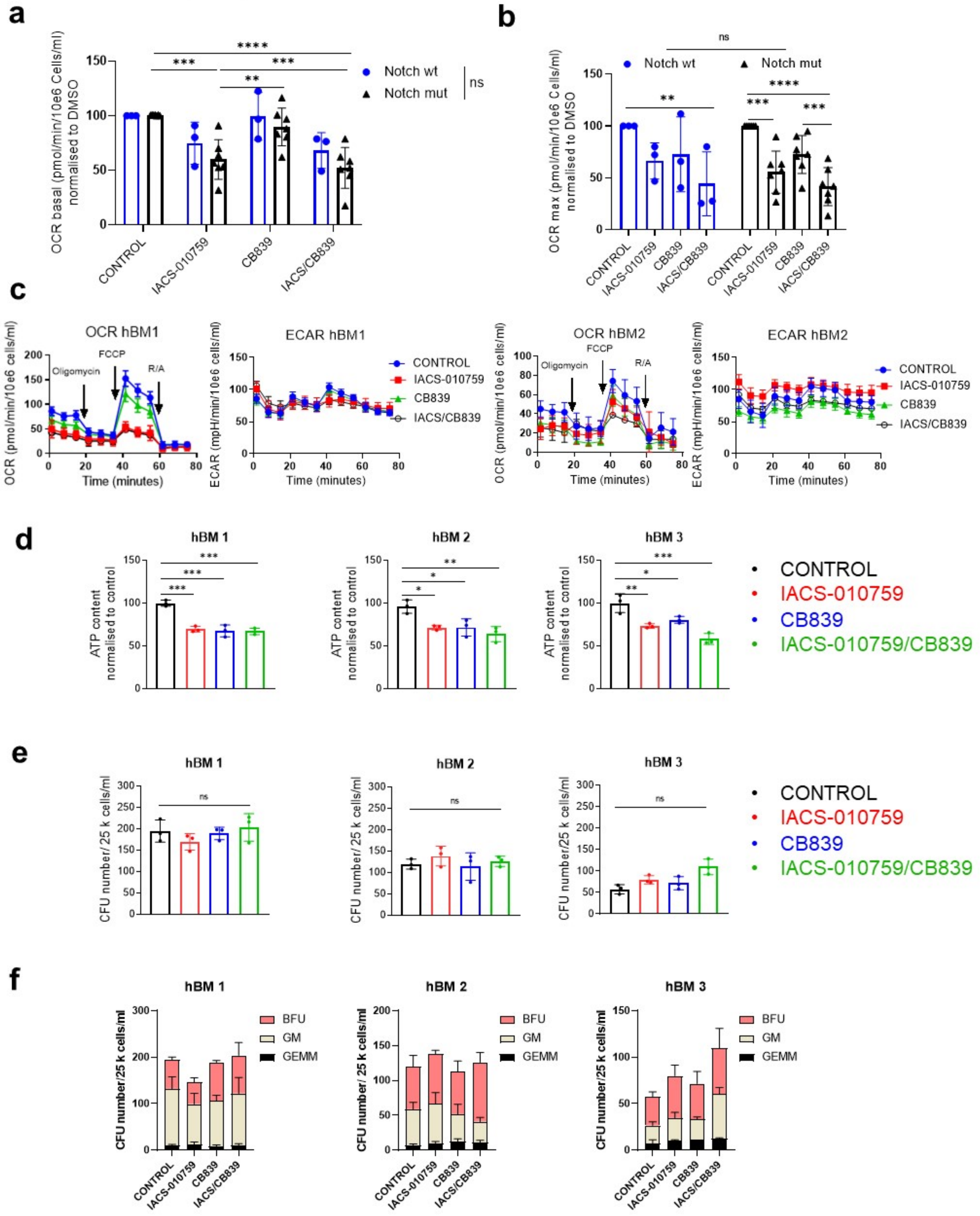

Supplementary Figure 16. CB-839 and IACS-010759 moderately inhibit OCR in healthy bone marrow cells without impacting colony forming unit ability

a) Basal OCR upon treatment with IACS-010759 at 10 nM, CB-839 1 uM, or combination of both compounds versus control in cell lines with *NOTCH1* mutation (n=7) and *NOTCH1* wt (n=3) (mean±SD, n=3 independent experiments with n=4 replicates per condition). Two-way ANOVA; P-values: ns-no significant, **=0.007; ***=0.0002; ****<0.0001

b) Maximal OCR response to treatment with IACS-010759 at 10 nM, CB839 1 uM, or combination of both compounds versus control in cell lines with *NOTCH1* mutation (n=7) and *NOTCH1* wt (n=3) (mean±SD, n=3 independent experiments with n=4 replicates per condition); Two-way ANOVA; P-values: ns-no significant, **<0.005; ***<0.001; ****<0.0001

c) Oxygen consumption rate (OCR) (left) and extracellular acidification rate (ECAR) (right) determined by Mito Stress Test in healthy donor derived bone marrow cells treated with 4 hr treatment with vehicle (control), 10 nM of IACS-010759, 12 hr treatment with 1 μM of CB-839; (n=2 independent hBM donors, with mean±SD, n=4 replicates per each condition);

d) ATP content analysis of human bone marrow cells derived from healthy donor following treatment with 1 μM CB-839; 10 nM IACS01759 or combination of both for 96 hr; normalised to DMSO-treated controls, from 3 independent bone marrow donors, as measured by CTG assay (mean±SD, n=3 technical replicates/condition);one-way ANOVA, P-values: *=0.033; **=0.0061; ***=0.0003;

e) Comparison of results for colony forming unit assay. Healthy bone marrow cells were obtained fresh from healthy bone marrow donors and seeded at the density of 1 million/ml for 24 hr with DMSO (CONTROL), 1 μM CB-839; 10 nM IACS01759 or combination of both, followed by collecting the cells and counting and seeding in Methylcellulose at the density of 25 k/ml for 12-14 days; colonies were counted manually from 3 independent dishes per consistency (mean±SD, n=3 replicates/treatment).ns-no significance; one-way ANOVA;

f) Presentation of colony units according the lineage and comparison across the treatments as described in (E) (mean±SD, n=3 replicates/treatment).

Supplementary Figure 17

Glucose labeling for CB839 and IACS-010759 in *NOTCH1* mutant

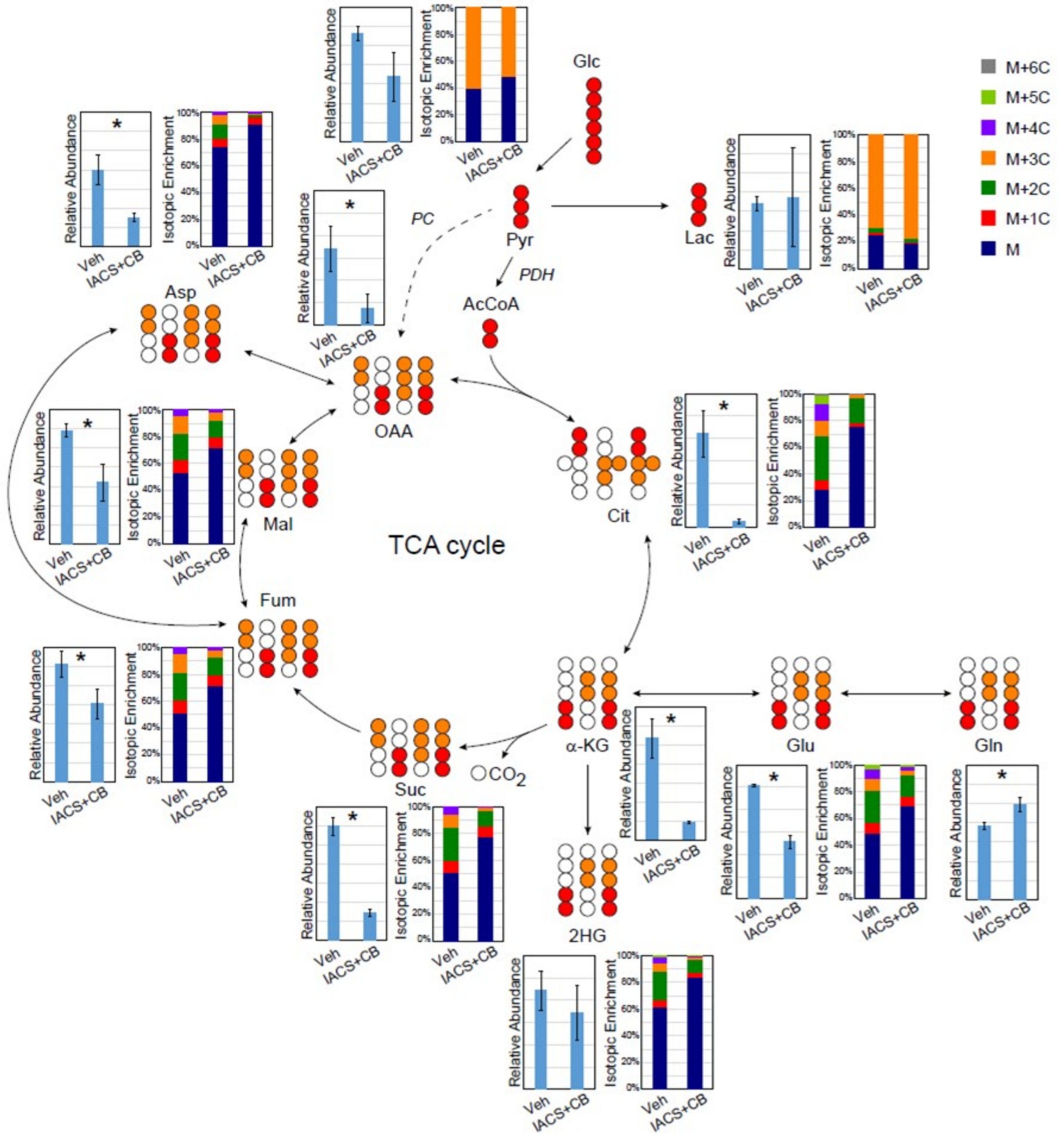

Supplementary Figure 17. Combination of IACS-010759 and CB-839 shows enhanced blockade of TCA cycle in NOTCH1 mutant T-ALL

Stable isotope-resolved metabolomics and ultra-performance liquid chromatography-tandem mass spectrometry analysis of NOTCH1-mutated T-ALL cell line PF-382 labelled with $^{13}C_6$-glucose and exposed to treatment with the combination of 10 nM IACS-010759 and 1 μM CB-839 for 12 h.

Supplementary Figure 18

a Glucose labeling for CB839 and IACS-010759 in *NOTCH1* wild type

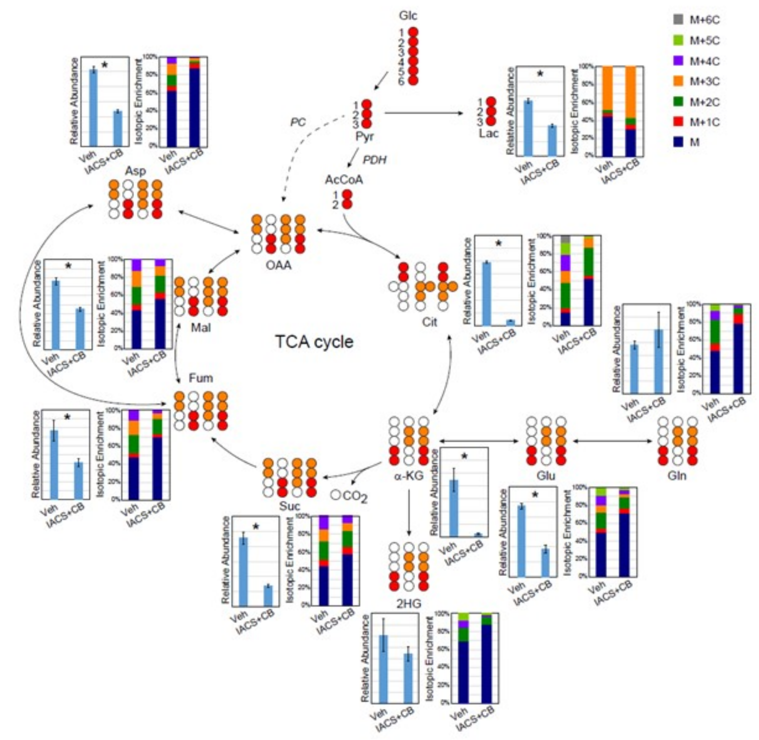

b Glutamine labeling for CB839 and IACS-010759 in *NOTCH1* wild type

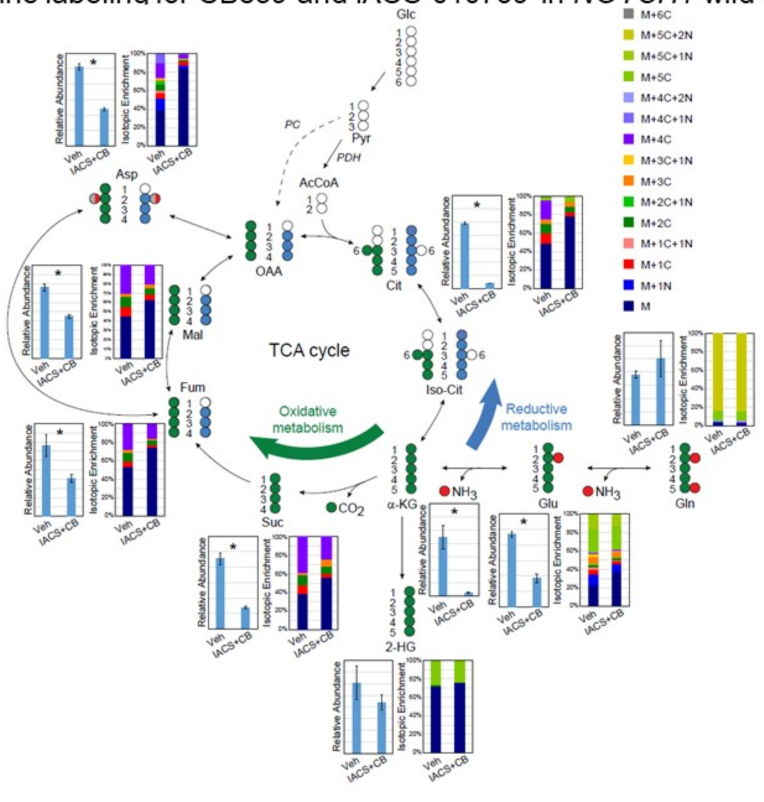

Supplementary Figure 18. Combination of IACS-010759 and CB-839 shows enhanced blockade of TCA cycle in NOTCH1 wild type T-ALL

a) Stable isotope-resolved metabolomics (SIRM) and ultra-performance liquid chromatography-tandem mass spectrometry (UPLC-MS/MS) analysis of *NOTCH1*-wild type T-ALL cell line SUP-T1 labelled with $^{13}C_6$-glucose and exposed to treatment with the combination of 10 nM IACS-010759 and 1 µM CB-839 for 24 h.

b) SIRM and UPLC-MS/MS analysis of *NOTCH1*-wild type T-ALL cell line SUP-T1 labelled with $^{13}C_5$, $^{15}N_2$-glutamine and exposed to treatment with the combination of 10 nM IACS-010759 and 1 µM CB-839 for 24 h.

Supplementary Figure 19

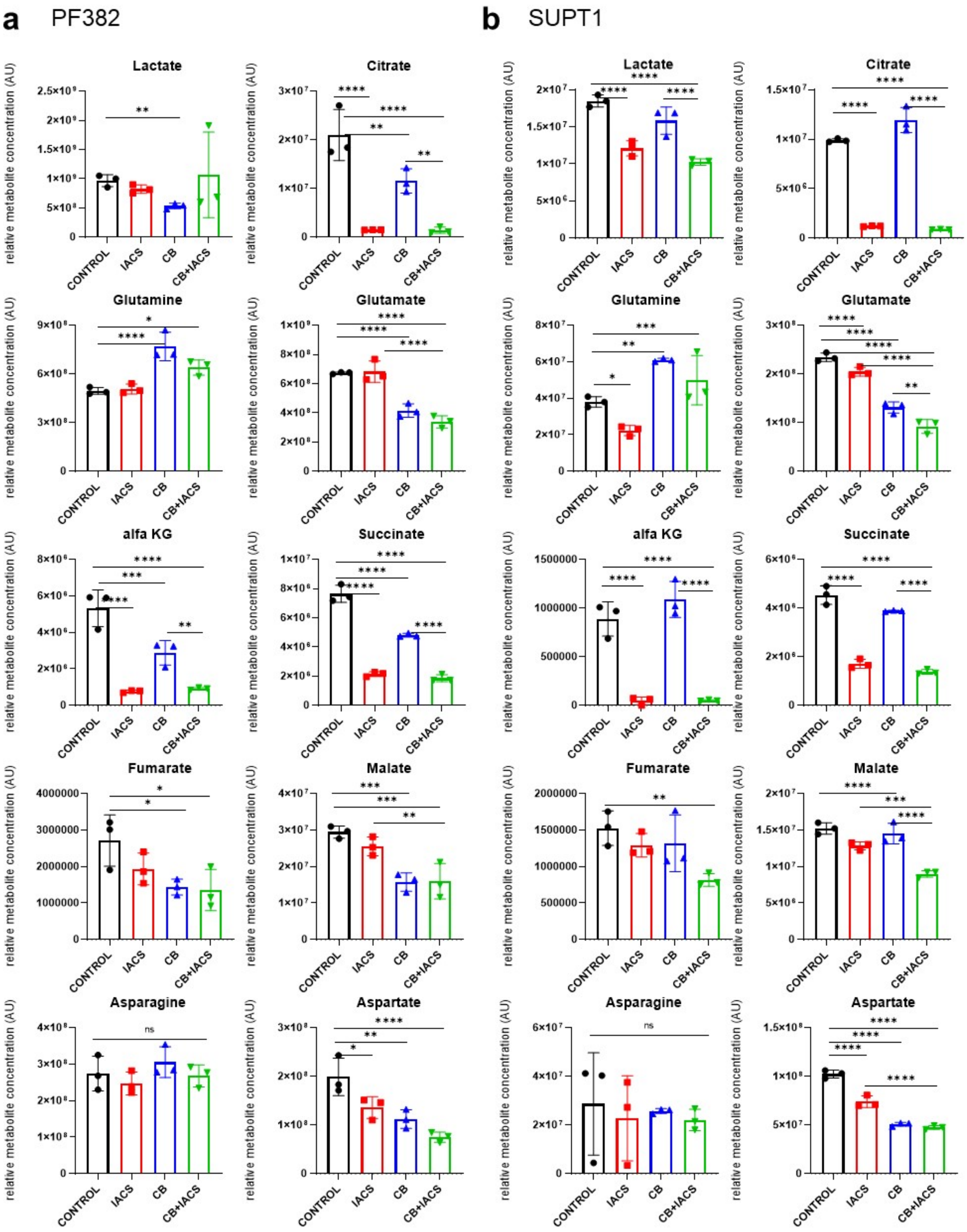

Supplementary Figure 19. SIRM and UPLC-MS/MS analysis indicate on-target blockade of both TCA cycle and glutaminolysis for T-ALL NOTCH1 mutant and NOTCH1 wild type cell line

a) SIRM and UPLC-MS/MS analysis of *NOTCH1*-mutated T-ALL cell line PF382 exposed to treatment with DMSO as CONTROL, 10 nM IACS-010759, 1 μM CB-839 or combination for 12 hrs (mean±SD, n=1 independent experiment with n=3 replicates per condition).

b) SIRM and UPLC-MS/MS analysis of *NOTCH1*-wild type T-ALL cell line SUP-T1 exposed to treatment with DMSO as CONTROL, 10 nM IACS-010759, 1 μM CB-839 or combination for 24 hrs (mean±SD, n=1 independent experiment with n=3 replicates per condition). P-values: *=0.0176; **=0.001; ***=0.0001; ****<0.0001, ns-no significant; one-way ANOVA.

Supplementary Figure 20

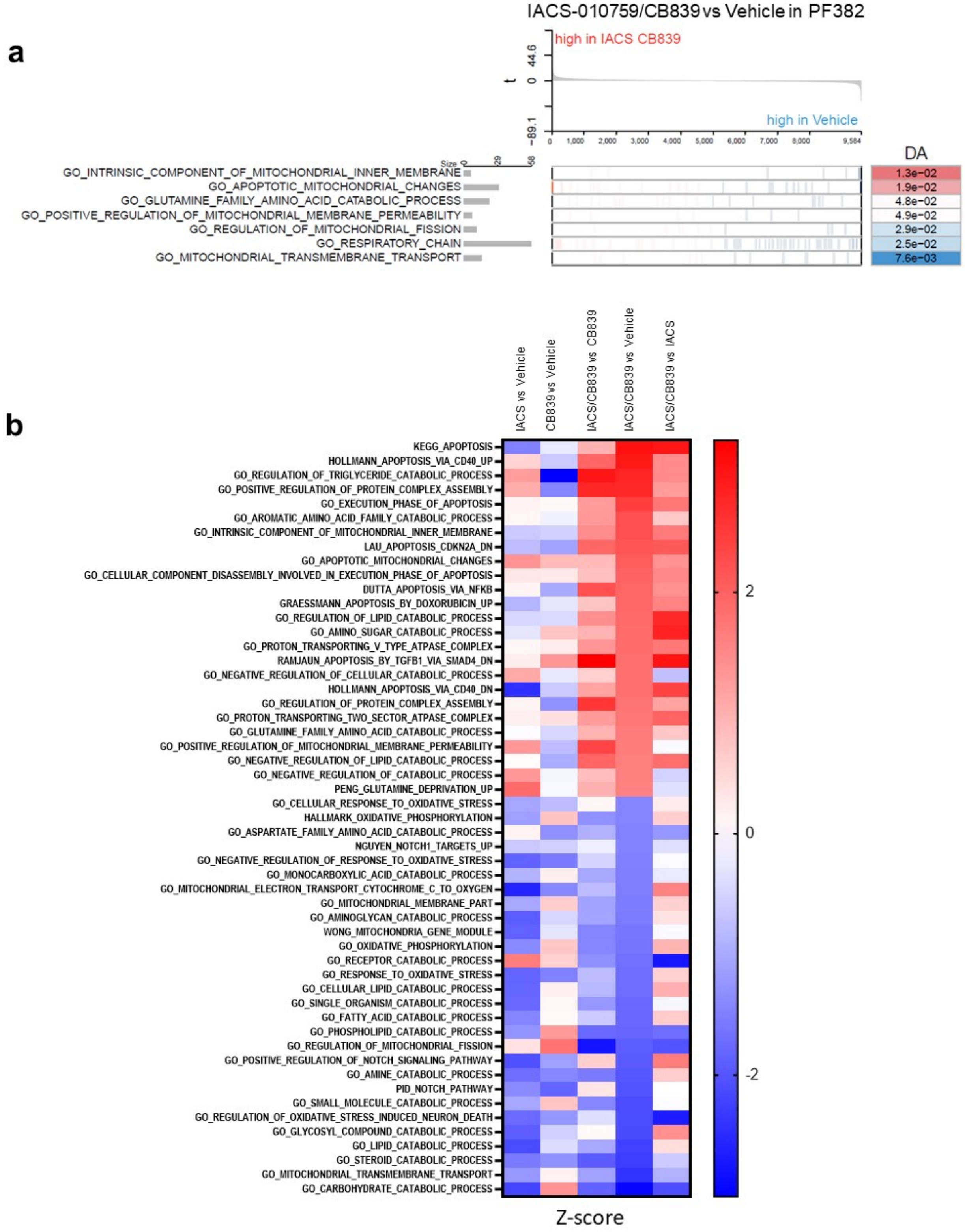

Supplementary Figure 20. Gen expression analysis indicates deep metabolic changes upon dual OxPhos- and GLS- blockade

a) Top: KEGG signaling pathways affected by IACS-010759 in combination with CB-839 in the *NOTCH1*-mutated cell line PF-382. Bottom: Gene Ontology analysis of genes affected by combination of IACS-010759 and CB-839.

b) Heatmap for gene set enrichment analysis (GSEA) displays differences in enrichment of genes related to glutamine metabolism, mitochondria translation and transcription, respiration and apoptosis analyzed by KEGG and displayed as a z-score affected by IACS-010759, CB-839 or combination of both compounds in the *NOTCH1*-mutated cell line PF-382.

Supplementary Figure 21

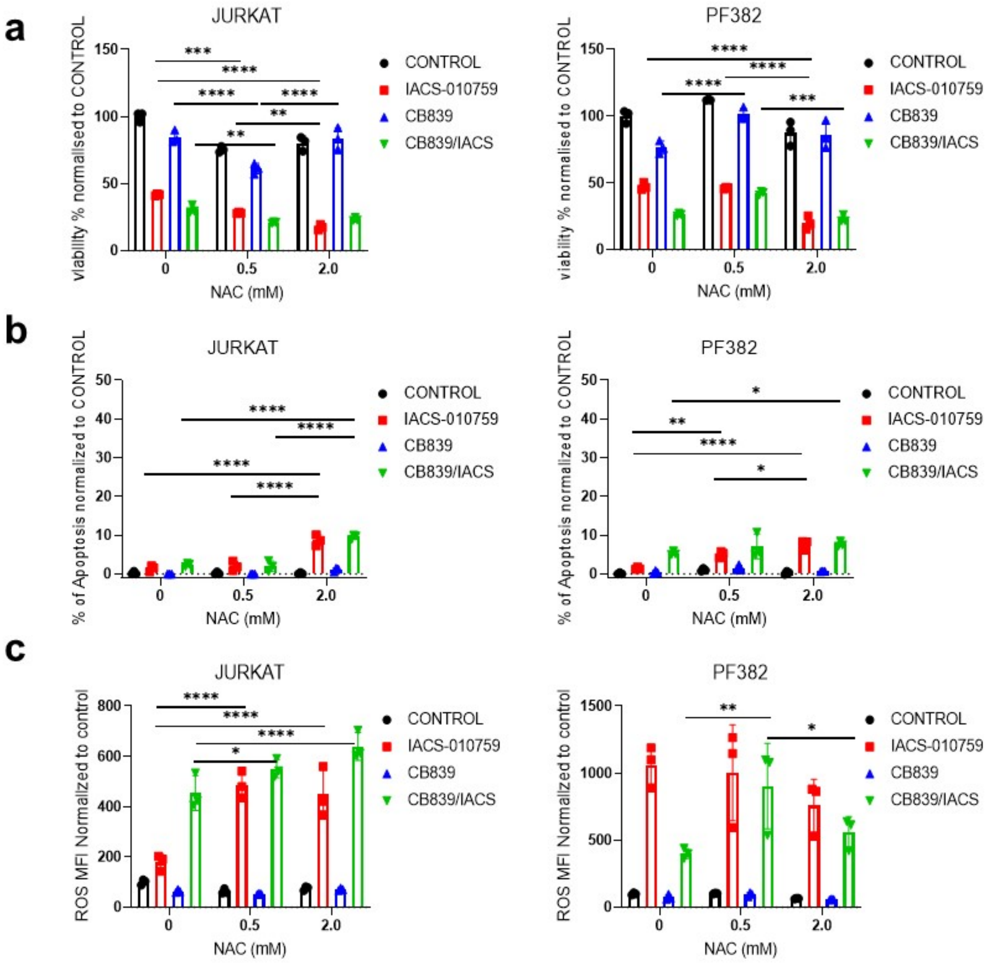

Supplementary Figure 21. N-acetylcysteine administration rescues blockade upon CB-839 but can't compensate metabolic changes upon dual metabolic inhibition of OxPhos and GLS

a) Viability analysis of NOTCH1-mutated T-ALL cell lines JURKAT and PF382, following treatment with 1 µM CB-839; 10 nM IACS01759 or combination of both for 72 hr; in presence of N-Acetylocysteine (NAC) at the concentration of 0.5 or 2.0 mM respectively, normalised to DMSO-treated controls, from 3 independent experiments (by flow cytometry); two-way ANOVA; P-values: **=0.0083; ***=0.00031; and ****<0.0001,

b) Apoptosis evaluation by Annexin V-assay from the experiment described in (A). two-way ANOVA; P-values: *=0.023; **=0.0011; ****<0.0001,

c) ROS evaluation as expressed by H2DCFDA mean fluorescence intensity (MFI) measured by flow cytometry normalised to CONTROL (cells treated with DMSO) as described in (A). All graphs show mean values, and error bars represent standard deviations, n=3 independent experiments. two-way ANOVA; P-values: *=0.03; **=0.0022; ****<0.0001.

Supplementary Figure 22

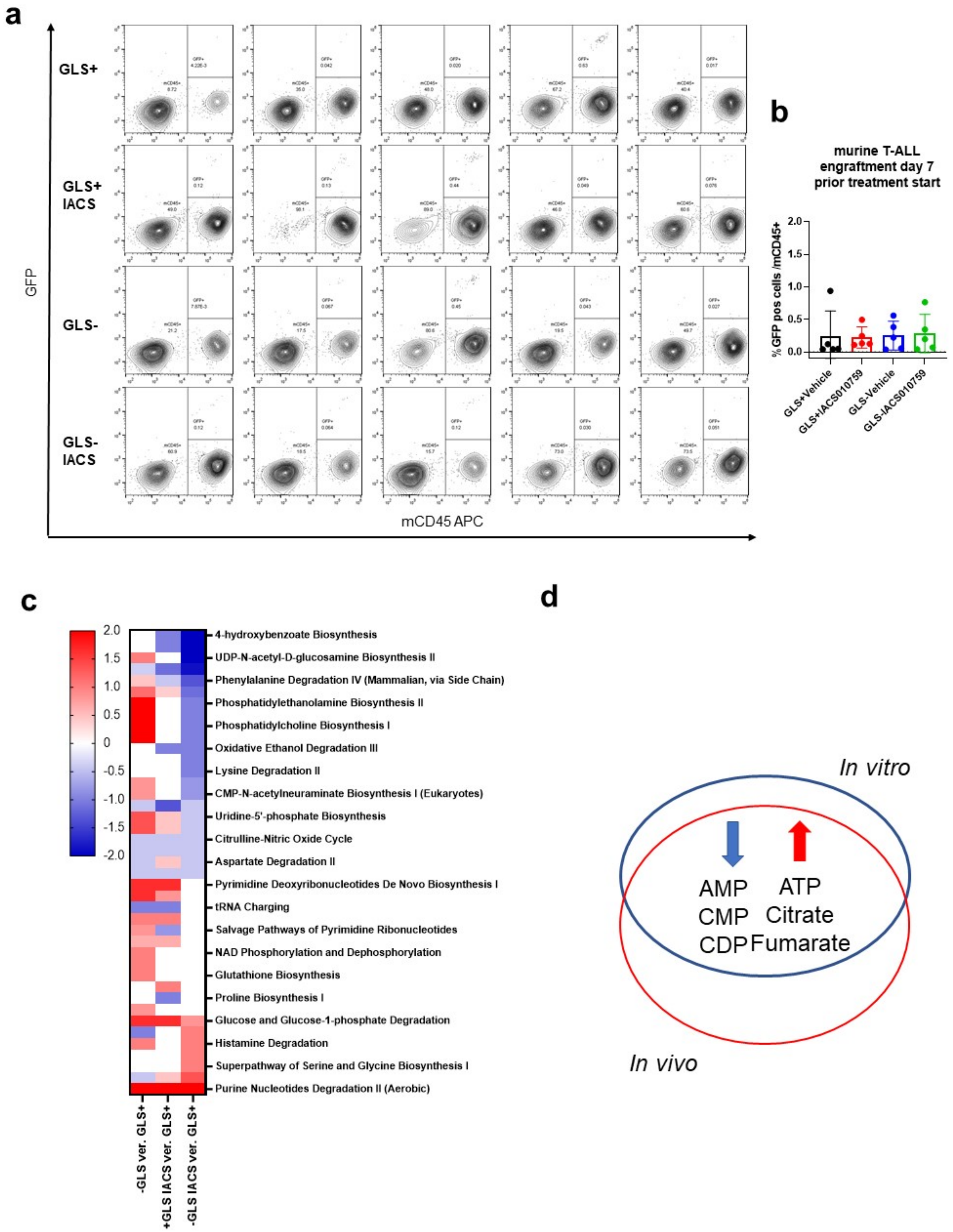

Supplementary Figure 22. Flow cytometry analysis indicate presence of circulating tumor cells prior initiation of treatment in mice harboring Notch1 mutated murine T-ALL leukemia, that undergo profound metabolic changes following treatment initiation.

a) Flow cytometry analysis of murine blood on day 7 post transplantation. After gating out debris and dead cells (DAPI+), double positive: GFP+, mCD45+ leukemic cells were identified on a GFP (FITC) (y-axis)- vs mCD45 (APC) (x-axis)-gated contour plot.

b) The average level of engraftment in BL6 mice harboring GLS fl/fl leukemia prior to treatment initiation: % of gated double positive cells were analyzed and divided by % of all mCD45+ cells and multiplied by 100% to obtain normalized leukemic engraftment, followed by mice randomization into 4 groups: GLS+, GLS+IACS-010759, GLS-, GLS-IACS010759 respectively (mean±SD, n=5 independent mice/ treatment group).

c) Heatmap of mass spectrometry analysis of metabolites found in PB of mice transplanted with murine NOTCH1-mutated T-ALL cells after 5 days of treatment with vehicle; IACS-010759; tamoxifen to induce GLS knockout, or combination of both tamoxifen and IACS-010759, (n=3 for each treatment). Results are expressed as a mean log of fold-change ratio over the level of metabolites measured in mice treated with vehicle;

d) Panel of common metabolites regulated in same way *in vivo* and *in vitro*.

Supplementary Figure 23

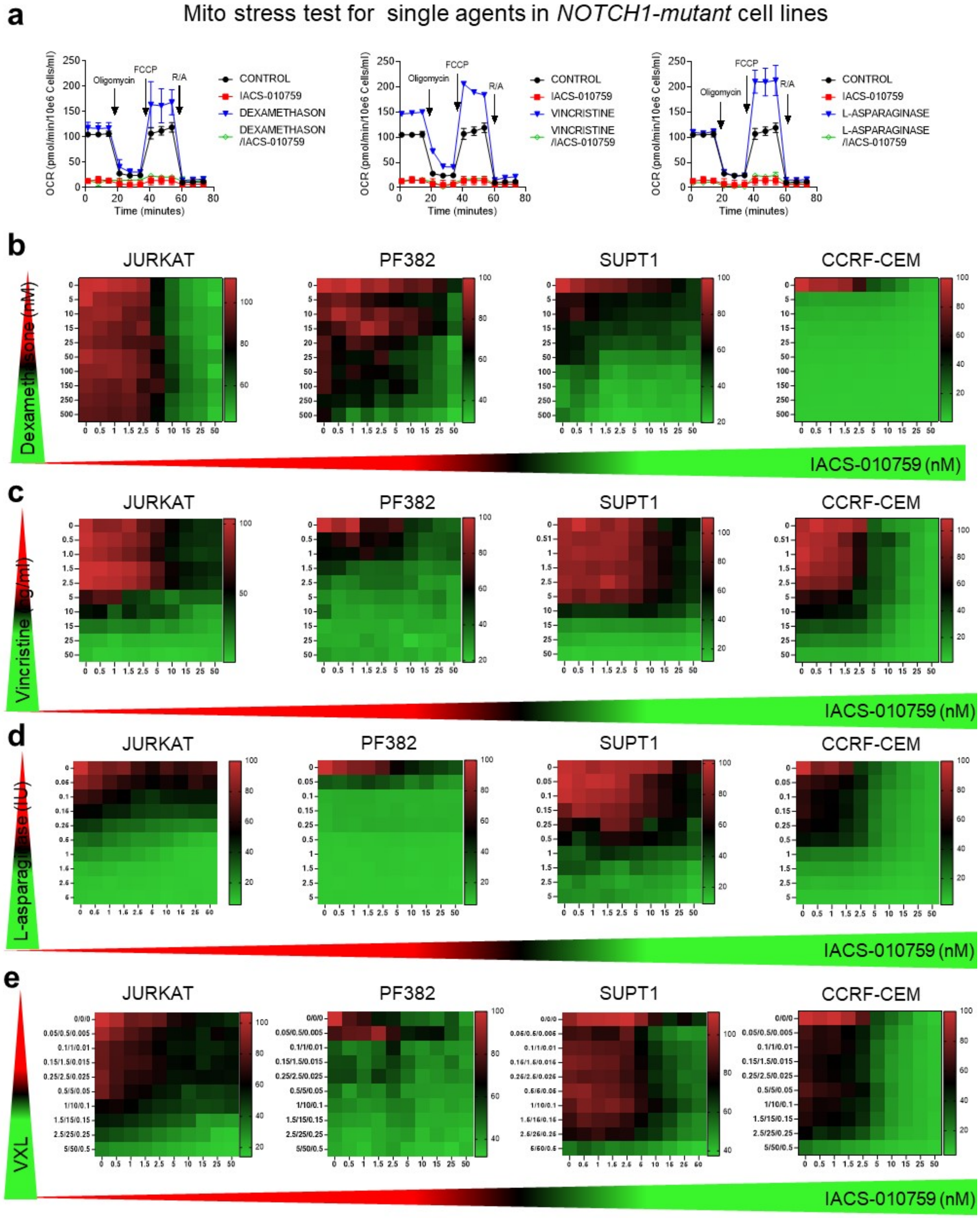

Supplementary Figure 23. IACS-010759 in combination with VXL produces viability reduction at low nanomolar concentrations overcoming intrinsic Dexamethasone resistance

a) Representative graphs of OCR measured by Mito Stress Test for the NOTCH1-mutated T-ALL cell line KOPT-K1 treated with (from left to right): dexamethasone, and/or IACS-010759; vincristine and/or IACS-010759, and L-asparaginase and/or IACS-010759 (mean±SD, n=3 independent experiments with n=4 replicates per condition).

b) Analysis of cell viability under treatment with increasing doses of IACS-010759 (0-50 nM) and dexamethasone (0-370 nM), in the indicated T-ALL cell lines;

c) Analysis of cell viability under treatment with increasing doses of IACS-010759 (0-50 nM) and vincristine (0-50 ng/mL), in the indicated T-ALL cell lines;

d) Analysis of cell viability under treatment with increasing doses of IACS-010759 (0-50 nM) and L-asparaginase (0-5 IU), in the indicated T-ALL cell lines;

e) Analysis of cell viability under treatment with increasing doses of IACS-010759 (0-50 nM) and VXL (0-15 ng/mL vincristine, 0-150 nM dexamethasone, 0-1.5 IU L-asparaginase) in the indicated T-ALL cell lines. Each square of the concentration matrix represents a mean value, normalized to that of DMSO-treated controls, from 5 independent experiments, as measured by CTG assay.

Supplementary Figure 24

a Mito stress test for chemotherapy and IACS-010759 in *NOTCH1* wild type cell lines

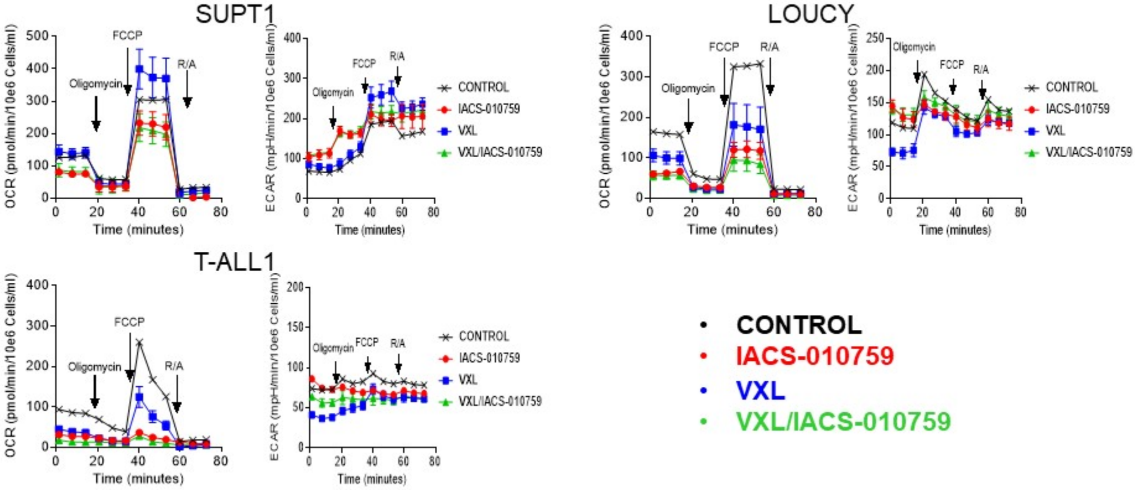

b Mito stress test for chemotherapy and IACS-010759 in *NOTCH1* mutated cell lines

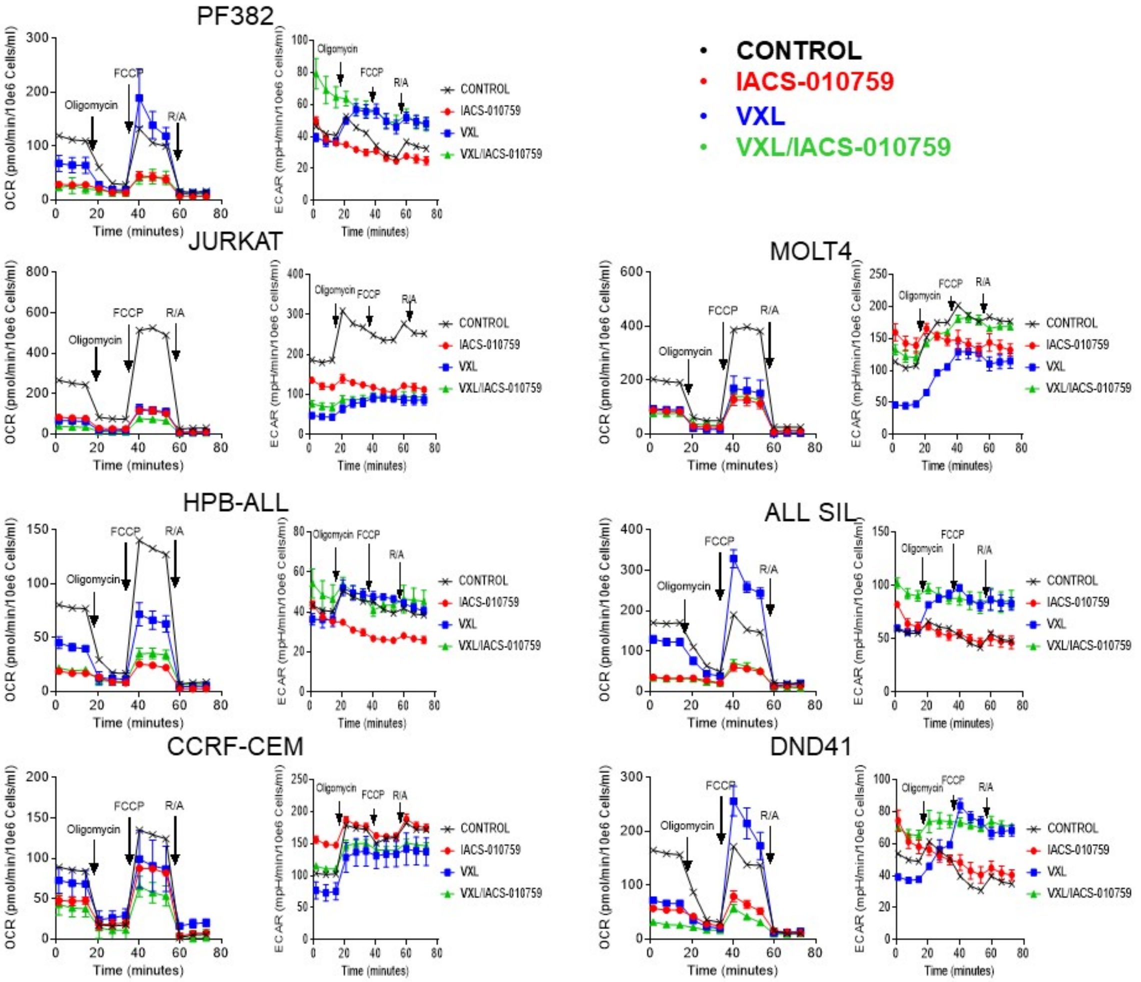

Supplementary Figure 24. VXL and IACS-010759 combination produces deeper reduction of OCR following VXL-driven upregulation of OCR in both NOTCH1 wild type and mutant T-ALL cell lines

a) Representative graphs of OCR and ECAR response determined by Mito Stress Test in NOTCH1-wild type T-ALL cell lines after treatment with DMSO, VXL, IACS-010759 and combination of VXL and IACS-010759. (mean±SD, n=3 independent experiments with n=4 replicates per condition)

b) Representative graphs of OCR and ECAR response upon treatment with DMSO, VXL, IACS-010759 and combination of IACS-010759 and VXL determined by Mito Stress Test in NOTCH1-mutated T-ALL cell lines in response to treatment with VXL and/or IACS-010759 (mean±SD, n=3 independent experiments with n=4 replicates per condition).

Supplementary Figure 25

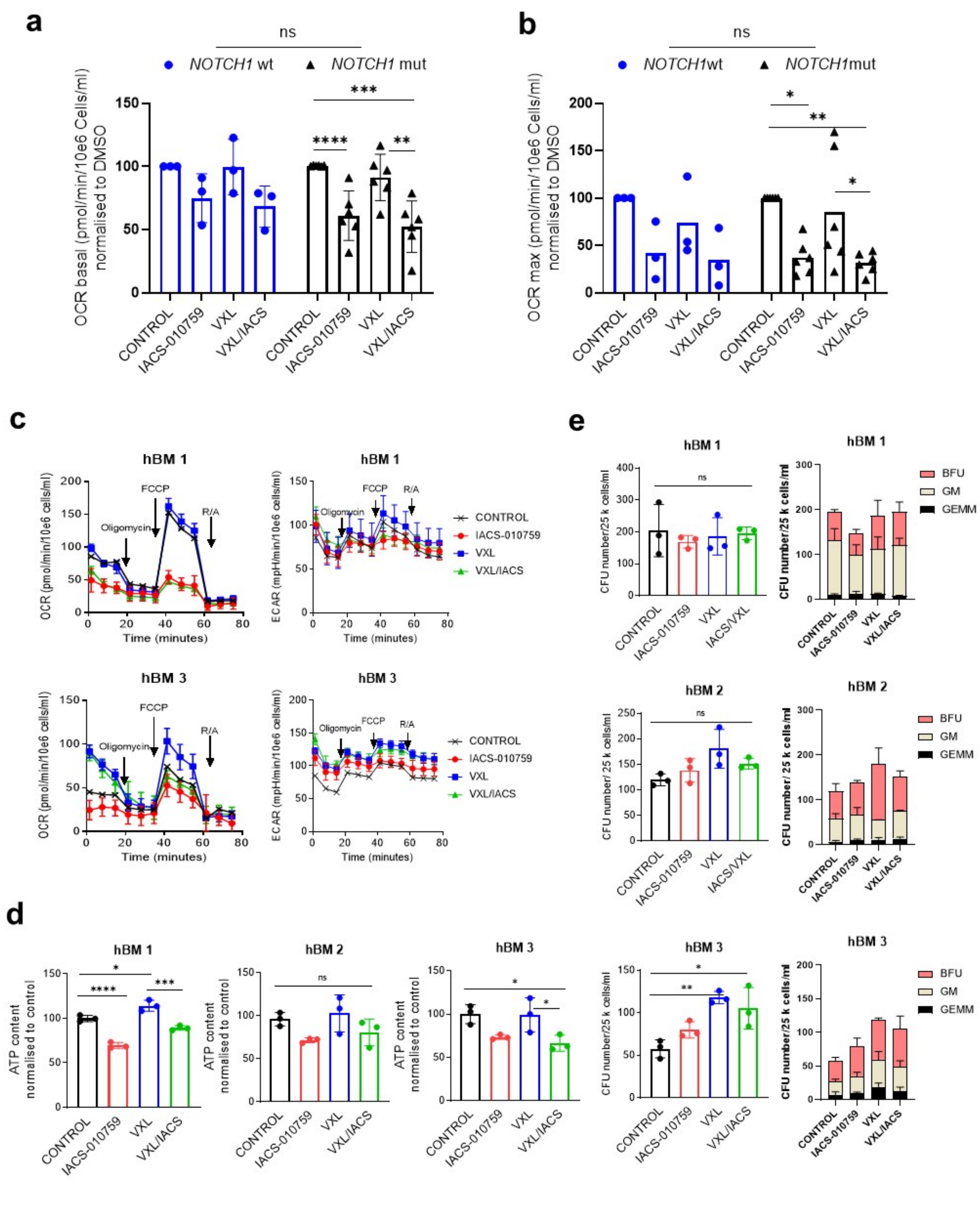

Supplementary Figure 25. IACS-010759 and VXL reduce moderatelyoxygen consumption rate in healthy bone marrow cells but don't reduce colony forming unit capacity

a) Basal OCR represented as summaries of 6 cell lines with NOTCH1 mutation and 3 cell lines with NOTCH1 wild type subjected to treatment with IACS-010759 at 10 nM, VXL, or combination of both compounds versus control (DMSO) (mean±SD, n=3 independent experiment, with 4 replicates per each treatment condition); Two-way ANOVA; p -value: ns-no significant, *=0.0208; **=0.0019; ***=0.0002; ****<0.0001;

b) Maximal OCR represented as summaries of 6 cell lines with NOTCH1 mutation and 3 cell lines with NOTCH1 wild type subjected to treatment with IACS-010759 at 10 nM, VXL, or combination of both compounds versus control (DMSO) (mean±SD, n=3 independent experiment, with 4 replicates per each treatment condition). Two-way ANOVA; p -value: ns-no significant, *=0.0106; **=0.0049;

c) Oxygen consumption rate (OCR) (left) and extracellular acidification rate (ECAR) (right) determined by Mito Stress Test in healthy donor derived bone marrow cells treated with 24 h treatment with vehicle (control), 10 nM of IACS-010759, VXL or its combination; (n=2 independent hBM donors, (mean±SD, n=4 replicates per each condition);

d) Analysis of ATP content of human bone marrow cells derived from healthy donor following treatment with VXL; 10 nM IACS01759 or combination of both for 96 hr; normalised to DMSO-treated controls, from 3 independent bone marrow donors, as measured by CTG assay; (mean±SD, n=3 replicates/condition); One-way ANOVA; p -value: ns-no significant, *=0.0409; ****<0.0001;

e) Comparison of results for the total number of colonies from colony forming unit assay (left) and presentation of colony units according to the lineage and comparison across the treatments (right); Healthy bone marrow cells were obtained fresh from healthy bone marrow donors and seeded at the density of 1 milion/ml for 24 hr with DMSO (CONTROL), 10 nM IACS01759, VXL or combination of both, following by collecting the cells and counting and seeding in Methylcellulose at the density of 25 k/ml for 12-14 days; colonies were counted manually from 3 independent dishes per consistency. (mean±SD); One-way ANOVA; p -value: *=0.016; **=0.0044;

Supplementary Figure 26

a Glucose labeling in *NOTCH1* mutated type

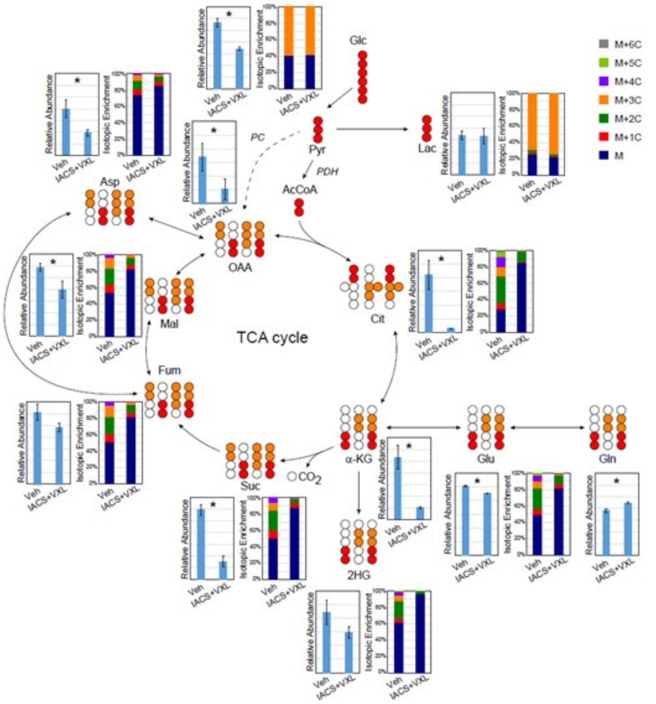

b Glutamine labeling in *NOTCH1* mutated type

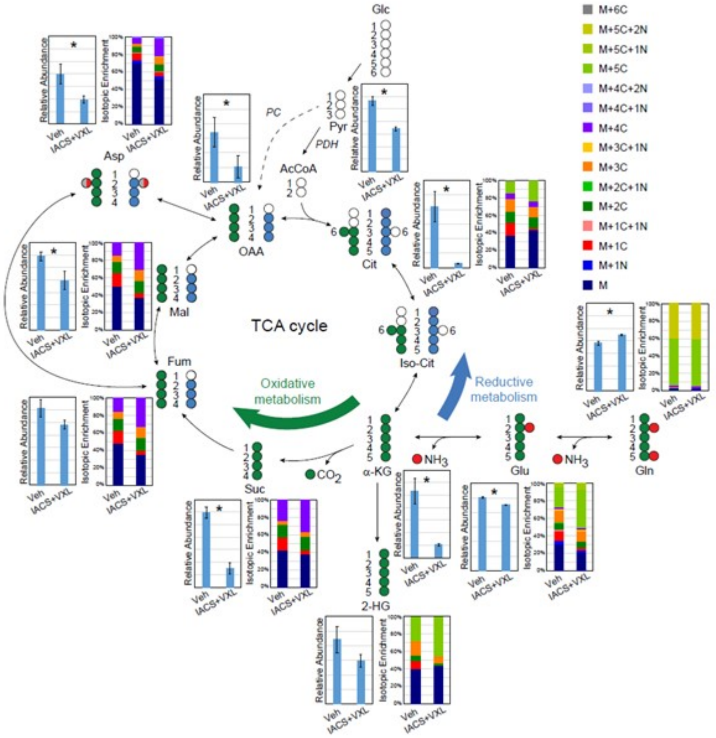

c

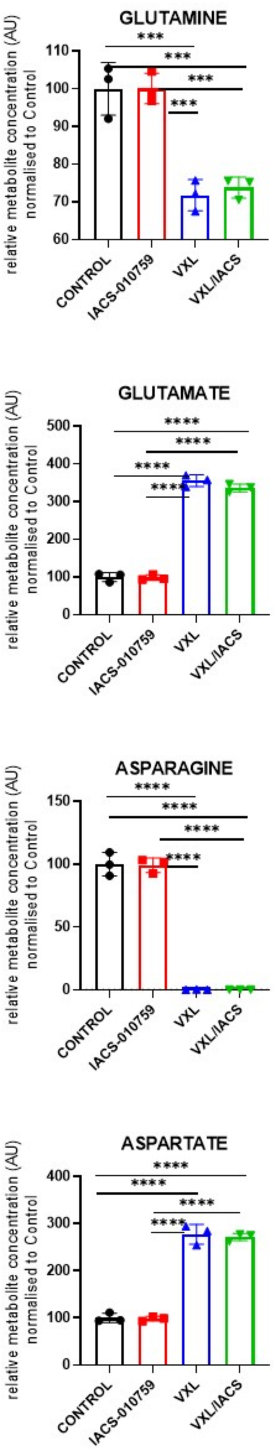

Supplementary Figure 26. SIRM and UPLC-MS/MS analysis indicate on-target blockade of both TCA cycle and glutaminolysis for T-ALL NOTCH1 mutant cell line PF382 upon combination of VXL and IACS-010759

a) Stable isotope-resolved metabolomics and ultra-performance liquid chromatography-tandem mass spectrometry analysis of *NOTCH1*-mutated T-ALL cell line PF-382 labelled with **(A)** $^{13}C_6$-glucose or

b) $^{13}C_5$, $^{15}N_2$-glutamine and exposed to treatment with the combination of 10 nM IACS-010759 and VXL for 12 h; (mean±SD, n=3 replicates/condition), two-tailed Student t-test.

c) Comparison of selected metabolites: glutamine, glutamate, Asparagine and aspartate measured in culture media collected from NOTCH1-mutated cell line PF382 cell culture after treatment with vehicle, IACS-010759, VXL or IACS-010759/VXL combination (mean±SD, n=1 independent experiment, with 3 replicates per each treatment condition). One-way ANOVA; p -value: ***=0.0004; ****<0.0001;

Supplementary Figure 27

a Glucose labeling in *NOTCH1* wild type

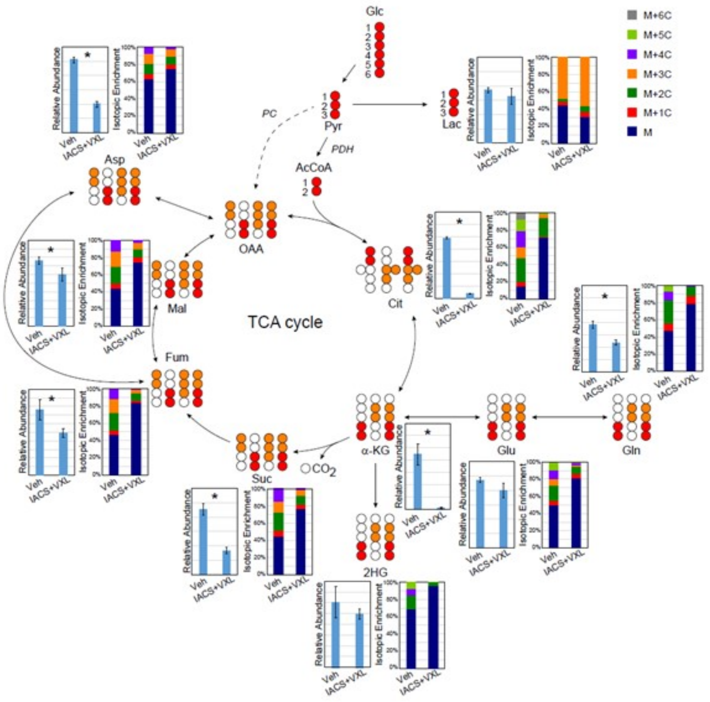

b Glutamine labeling in *NOTCH1* wild type

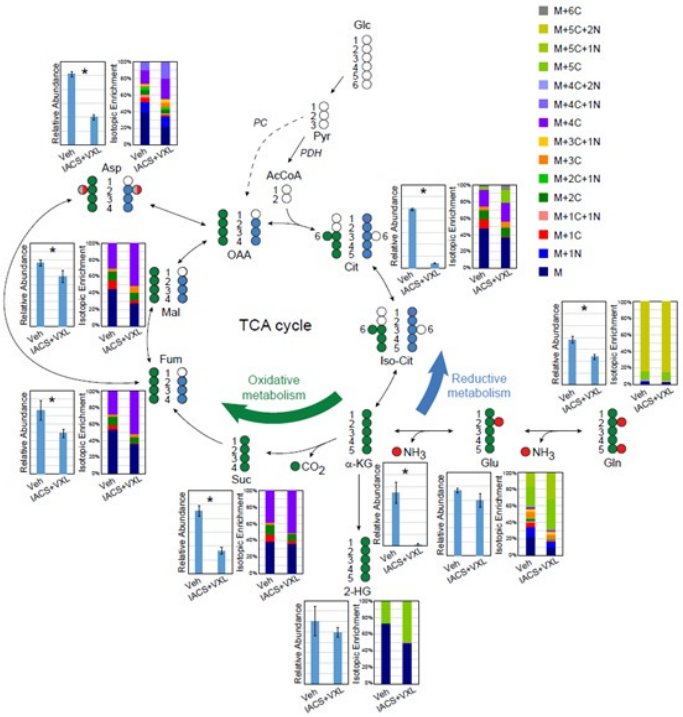

c

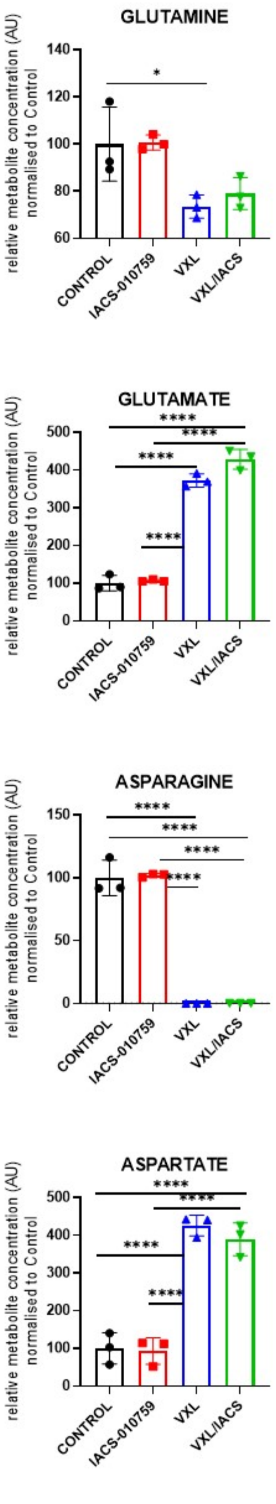

Supplementary Figure 27. SIRM and UPLC-MS/MS analysis indicate on-target blockade of both TCA cycle and glutaminolysis for T-ALL NOTCH1 wild type cell line SUP-T1 upon combination of VXL and IACS-010759

a) Stable isotope-resolved metabolomics and ultra-performance liquid chromatography-tandem mass spectrometry analysis of the NOTCH1-wild type T-ALL cell line SUP-T1 labelled with **(A)** 13C6-glucose or

b) 13C5, 15N2-glutamine and treated with the combination of 10 nM IACS-010759 and VXL for 24 hrs. (mean±SD, n=3 replicates/condition), two-tailed Student t-test.

c) Comparison of selected metabolites: glutamine, glutamate, Asparagine and aspartate measured in culture media collected from NOTCH1-wild type cell line SUPT1 cell culture after treatment with vehicle, IACS-010759, VXL or IACS-010759/VXL combination (mean±SD, n=1 independent experiment, with 3 replicates per each treatment condition). One-way ANOVA; p -value: *=0.0263; ****<0.0001;

Supplementary Figure 28

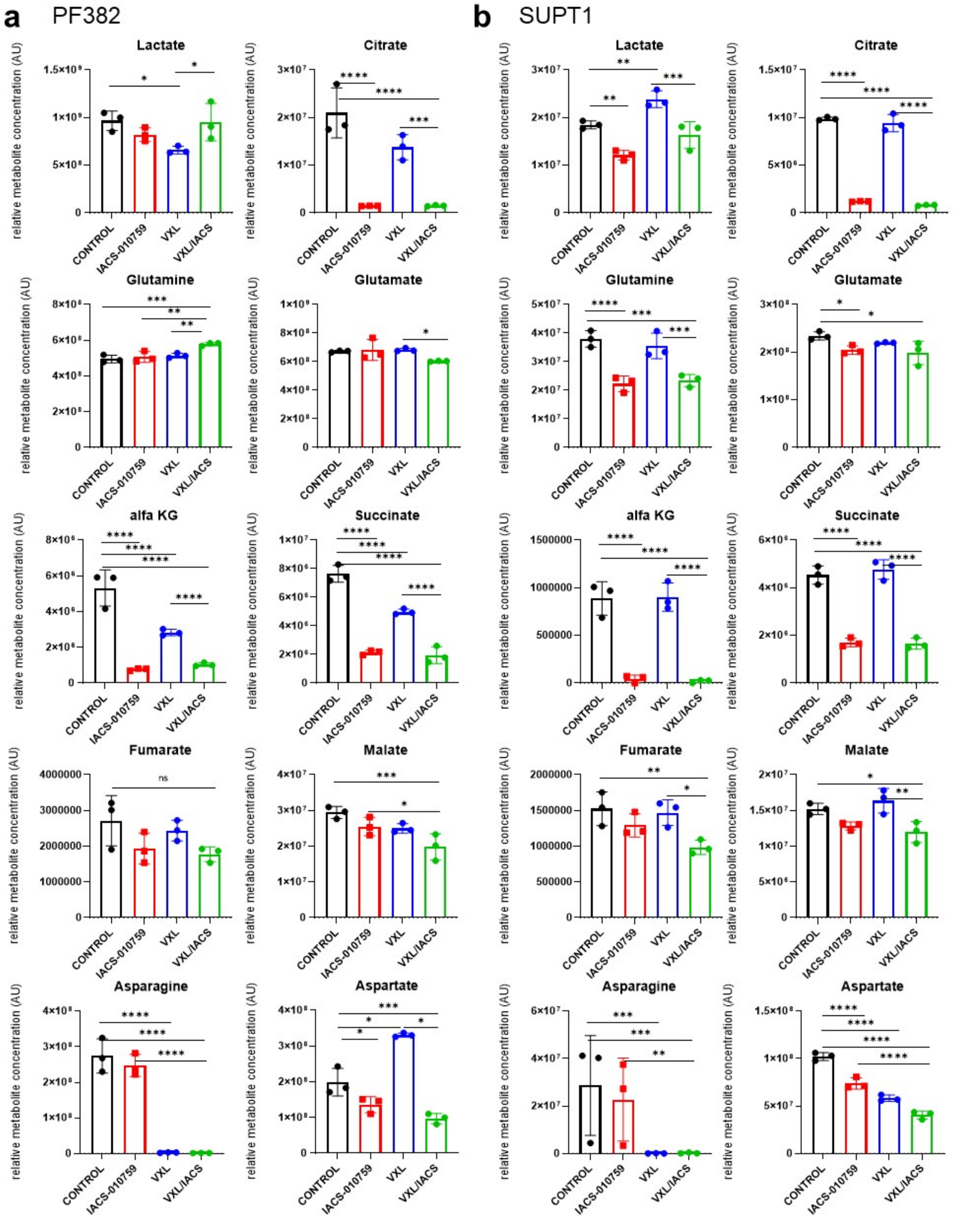

Supplementary Figure 28. SIRM and UPLC-MS/MS analysis indicate on-target blockade of both TCA cycle and glutaminolysis for T-ALL NOTCH1 mutant and NOTCH1 wild type cell line upon combination of VXL and IACS-010759

a) SIRM and UPLC-MS/MS analysis of *NOTCH1*-mutated T-ALL cell line PF382 exposed to treatment with DMSO as CONTROL, 10 nM IACS-010759, VXL or combination for 12 hrs (mean±SD, n=1 independent experiment with n=3 replicates per condition)

b) SIRM and UPLC-MS/MS analysis of *NOTCH1*-wild type T-ALL cell line SUP-T1 exposed to treatment with DMSO as CONTROL, 10 nM IACS-010759, VXL or combination for 24 hrs (mean±SD, n=1 independent experiment with n=3 replicates per condition);

P-values: *=0.0104; **=0.0043; ***=0.0002; ****<0.0001, ns-no significant; one-way ANOVA.

Supplementary Figure 29

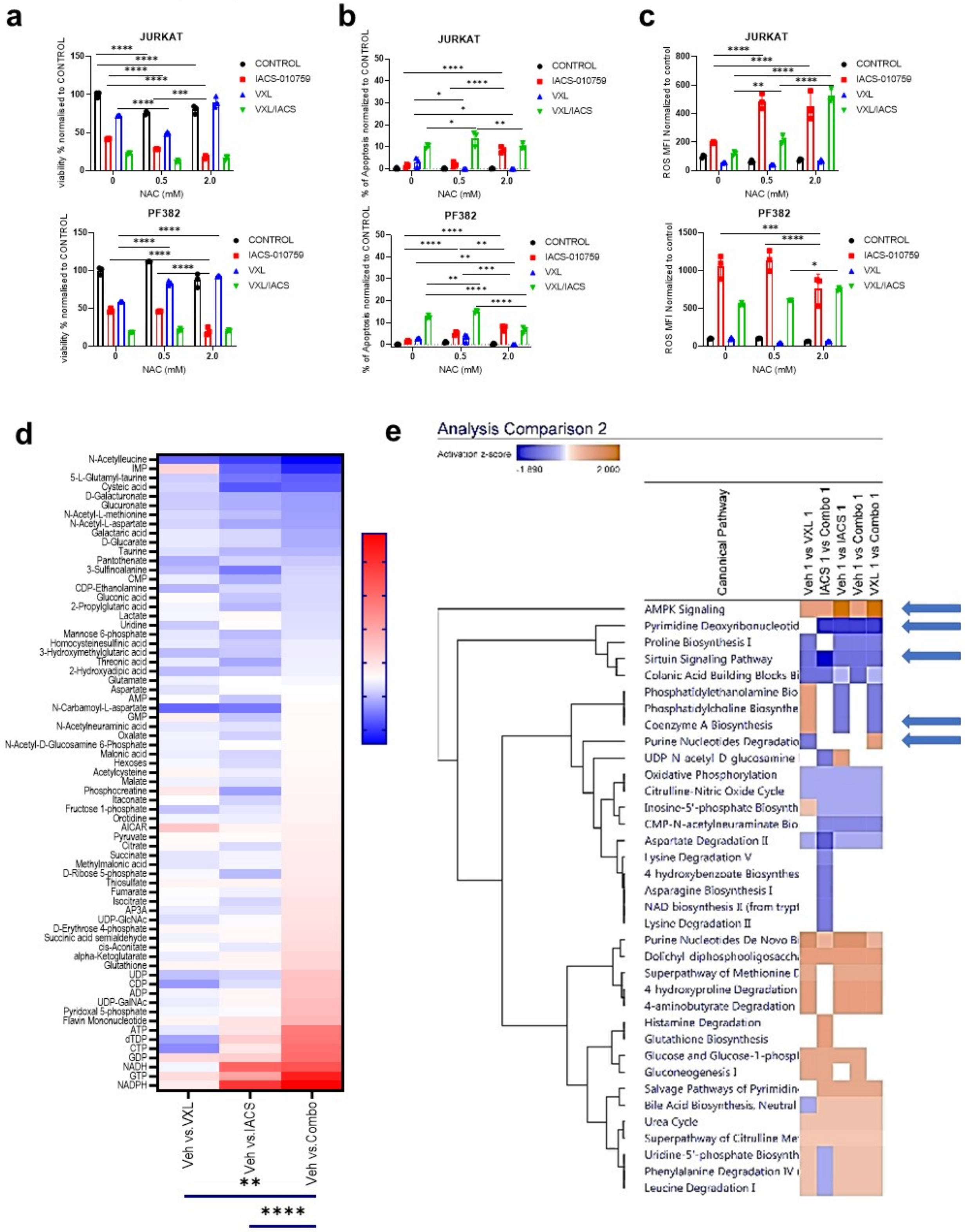

Supplementary Figure 29. Impact on cell growth inhibition and apoptosis induction of combined IACS-010759/VXL blockade *in vitro* cant be reversed by NAC, but leads to profound metabolic changes as shown in circulating leukemic cells *in vivo*

a) Viability analysis of NOTCH1-mutated T-ALL cell lines JURKAT and PF382, following treatment with 10 nM IACS01759, VXL or combination of both for 72 hrs; in presence of N-Acetylcysteine (NAC) at the concentration of 0.5 or 2.0 mM respectively, normalised to DMSO-treated controls (by flow cytometry);(mean±SD, n=3 independent experiments), two-way ANOVA; P-values: **=0.0083; ***=0.00031; and ****<0.0001,

b) Apoptosis evaluation by Annexin V-assay from the experiment described in (A); (mean±SD, n=3 independent experiments), two-way ANOVA; P-values: *=0.03; **=0.0096; ***=0.0008; ****<0.0001,

c) ROS evaluation as expressed by H2DCFDA mean fluorescence intensity (MFI) measured by flow cytometry normalised to CONTROL (cells treated with DMSO) as described in (A). (mean±SD, n=3 independent experiment, with 3 replicates per each treatment condition), two-way ANOVA; P-values: *=0.02; **=0.0099; ****<0.0001,

d) Heatmap of mass spectrometry analysis of metabolites found in PB of mice transplanted with murine Notch1-mutated PDX 80 T-ALL cells after 12 hr of treatment with vehicle; IACS-010759; VXL and combination of VXL and IACS-010759, (n=2 for each treatment). Results are expressed as a mean log of fold-change ratio over the level of metabolites measured in mice treated with vehicle;

e) Ingenuity Pathway Analysis of metabolites presented as heatmap showing the most important metabolism-related canonical pathways altered under VXL and chemical inhibition of complex I, analyzed by Ingenuity Pathway Analysis software (QIAGEN);

Supplementary Figure 30 Bone Marrow

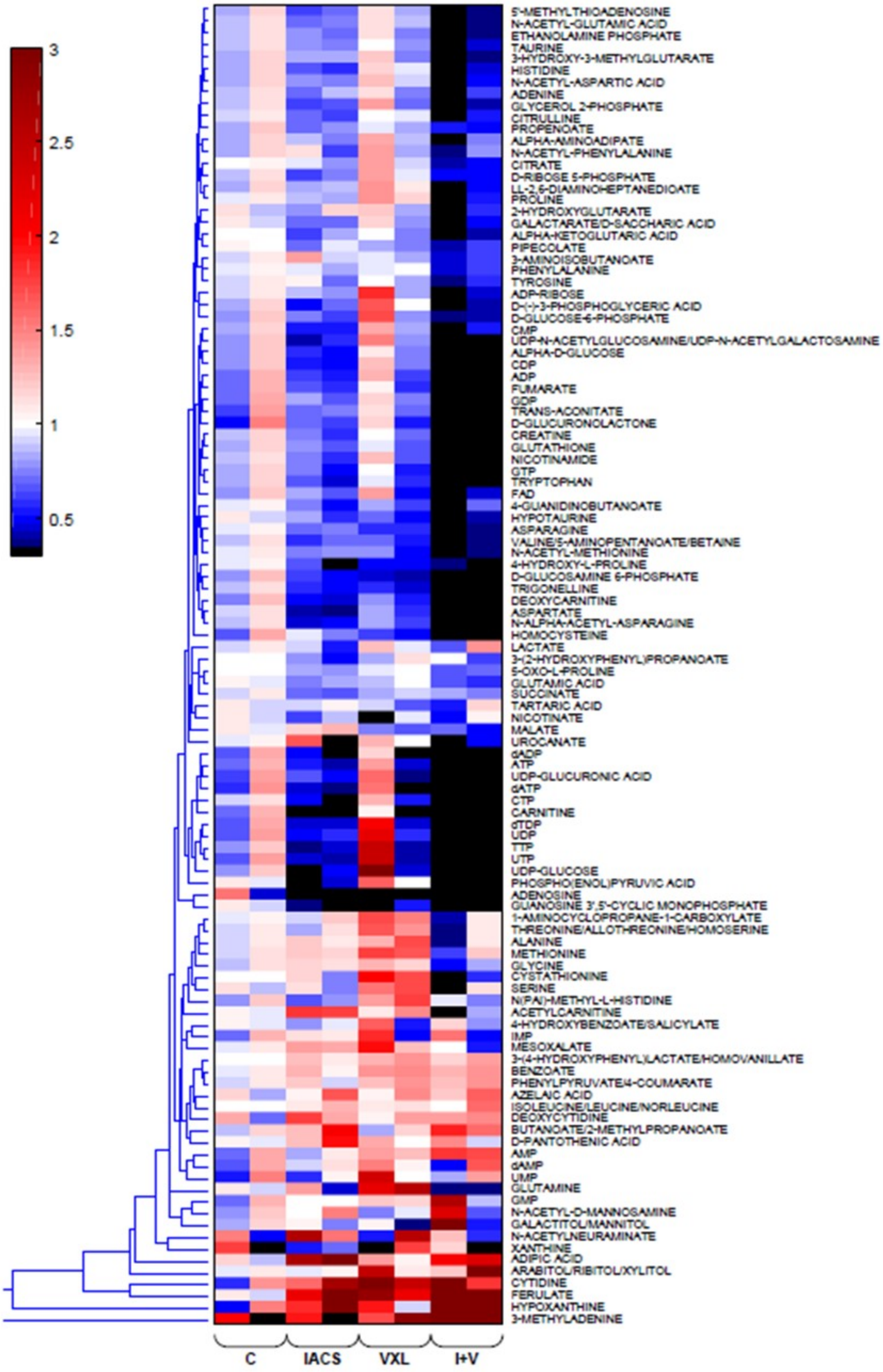

Supplementary Figure 30. Mass spectrometry analysis of bone marrow cells derived from mice harboring PDX 80 indicate profound changes upon IACS-010759 and VXL dual intervention

Heatmap of mass spectrometry analysis of metabolites in bone marrow of mice transplanted with NOTCH1-mutated PDX80 after 12 hrs of treatment with vehicle, IACS-010759, VXL, or the combination of IACS-010759 and VXL (n=2 for each treatment);

Supplementary Figure 31 Spleen

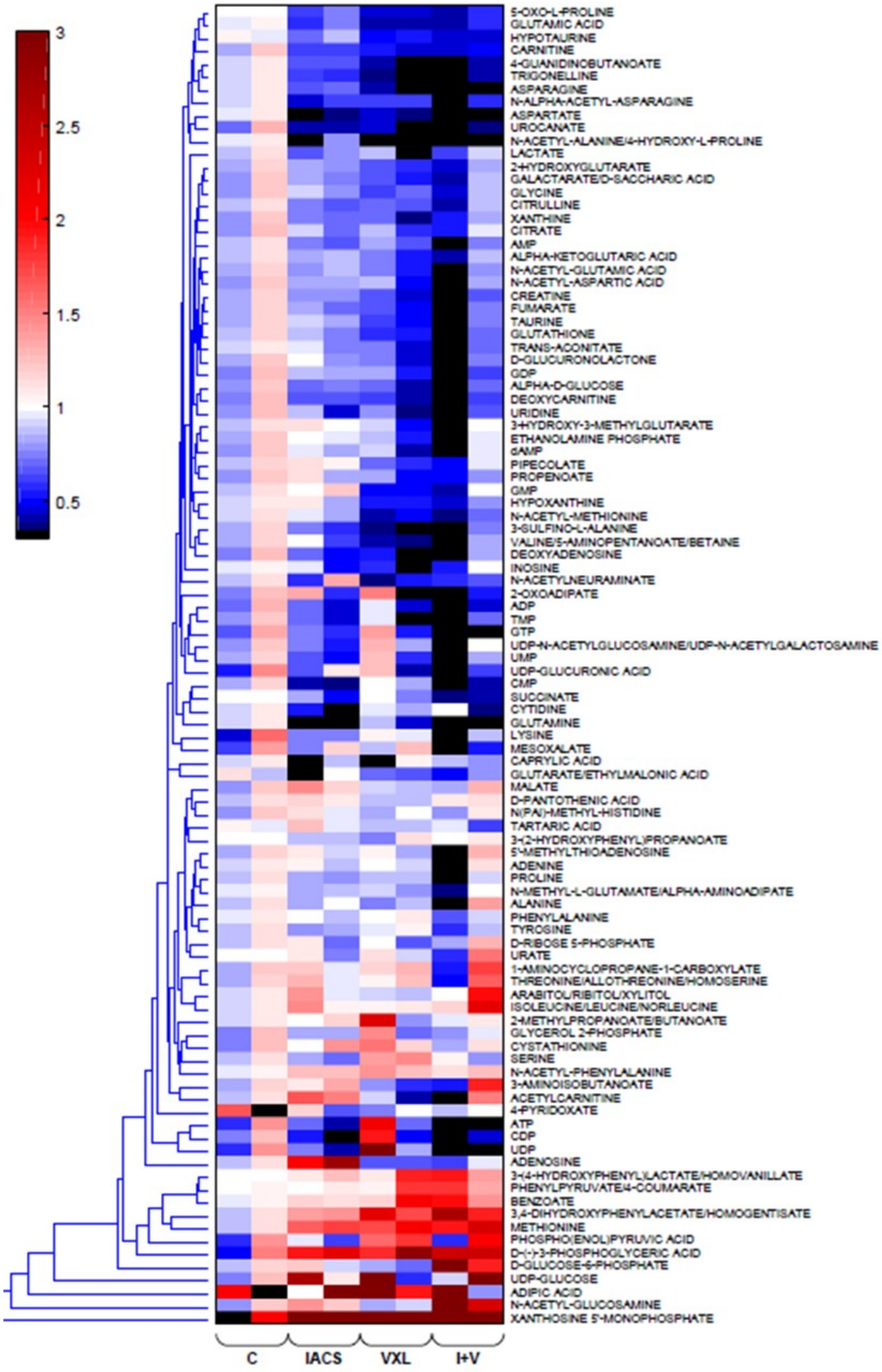

Supplementary Figure 31. Mass spectrometry analysis of spleen cells derived from mice harboring PDX 80 indicate profound changes upon IACS-010759 and VXL dual intervention

Heatmap of mass spectrometry analysis of metabolites in spleens of mice transplanted with NOTCH1-mutated PDX80 after 12 hrs of treatment with vehicle, IACS-010759, VXL, or the combination of IACS-010759 and VXL (n=2 for each treatment);

Supplementary Figure 32

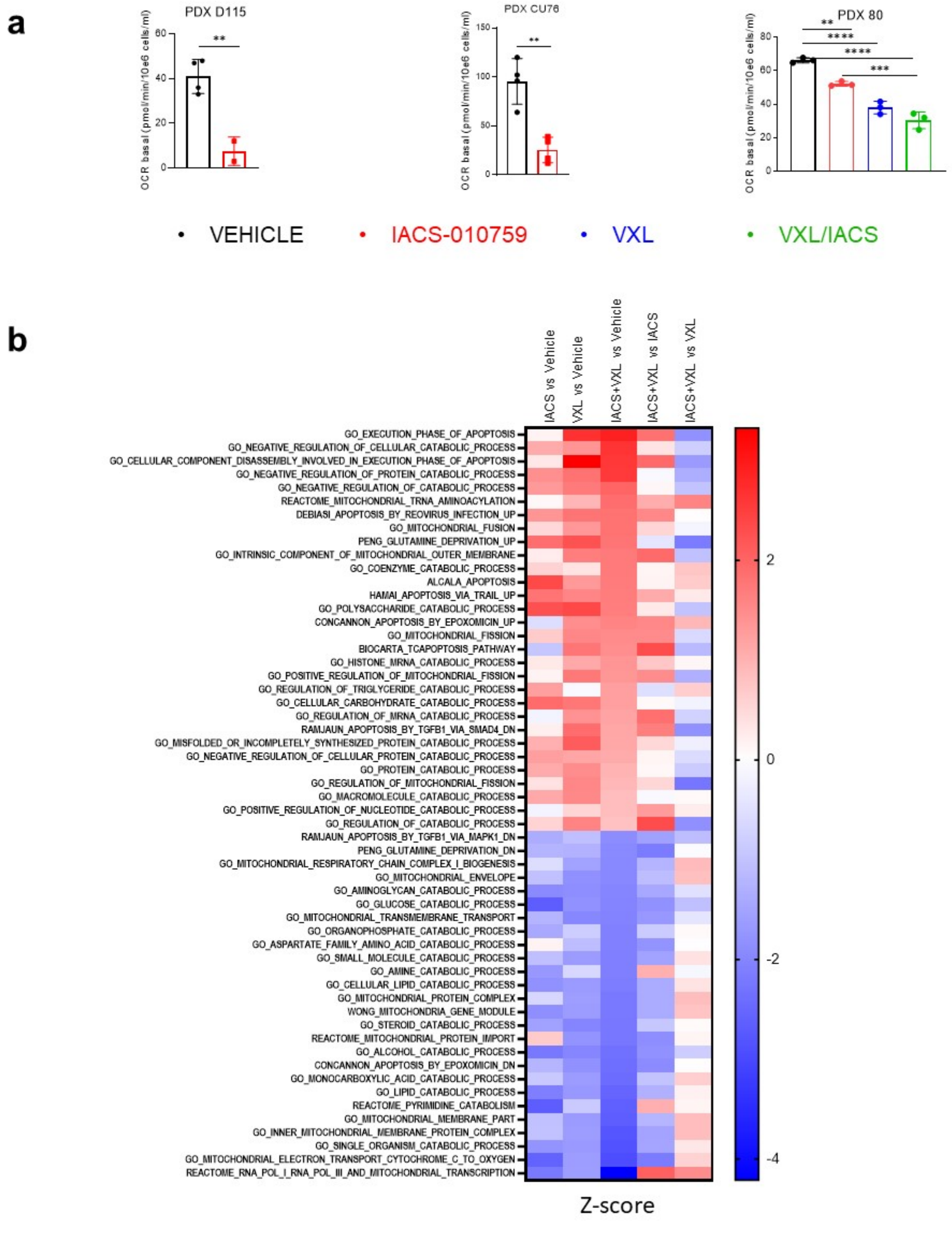

Supplementary Figure 32. *Ex vivo* OCR measurement confirms reduction of oxidative respiration upon VXL and IACS-010759 treatment, blocking biosynthesis and bioenergetic processes in mitochondria in T-ALL PDX model D115

a) Oxygen consumption rate (OCR) in spleen cells harvested from mice bearing D115, CU76, and PDX80 PDXs treated with vehicle (black), VXL (blue), IACS-010759 (red), or the combination of VXL with IACS-010759 (green) (mean±SD, each dot represents mean of 4 technical replicates for individual mice); P-values: **=0.0035; ***=0.0002; ****<0.0001, one-way ANOVA.

b) Heatmap for gene set enrichment analysis (GSEA) displays differences in enrichment of genes related to glutamine metabolism, mitochondria translation and transcription, respiration and apoptosis analyzed by KEGG and displayed as a z-score affected by IACS-010759, VXL or combination of both compounds in the NOTCH1-mutated cell line PF-382.

Supplementary Figure 33

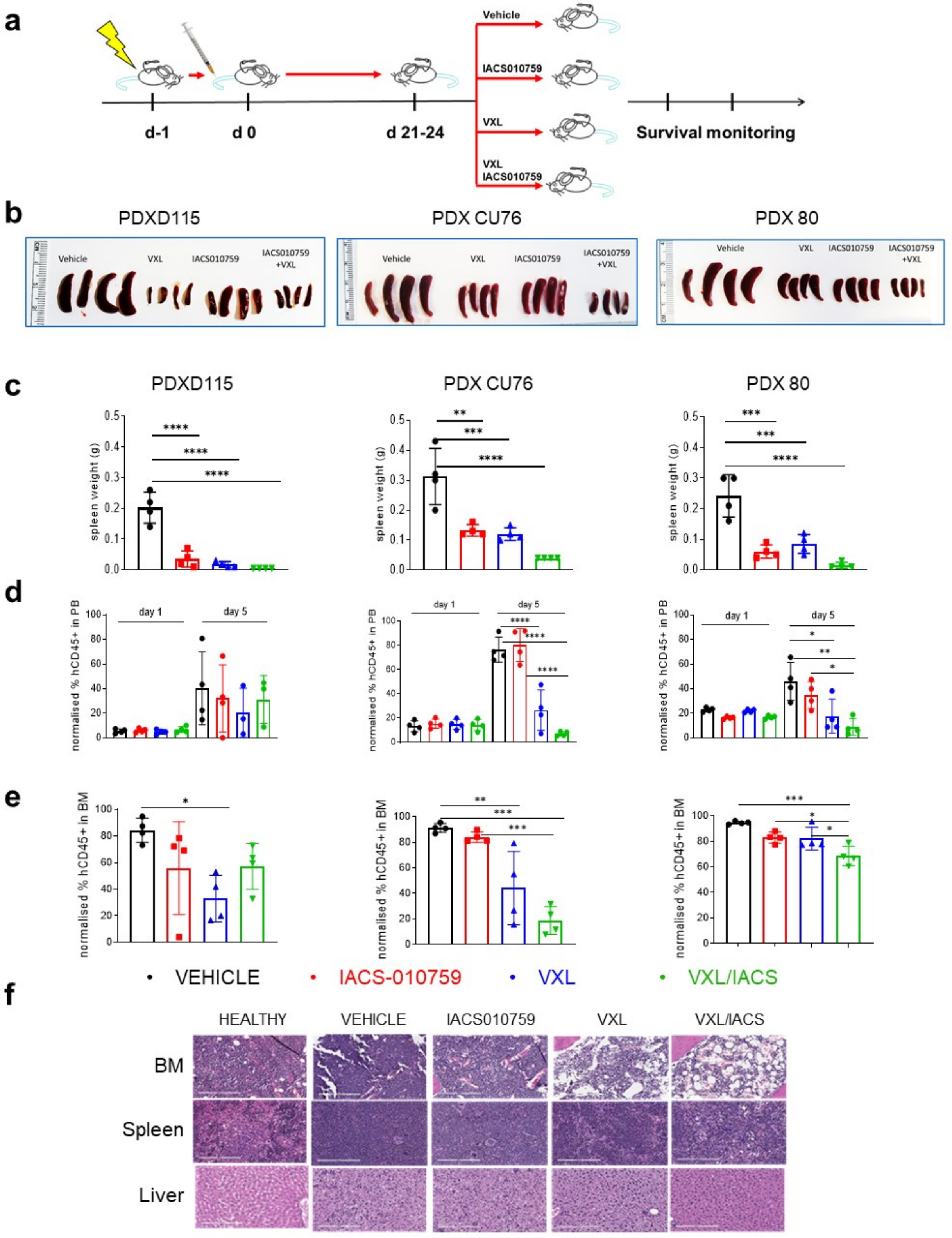

Supplementary Figure 33. Effects of VXL and OxPhos inhibition on tumor burden in T-ALL PDX models.

a) Schematic of study design using 3 PDXs transplanted into sub lethally irradiated NSG mice;

b) Size of spleens from mice transplanted with PDX D115, PDX CU76, and PDX 80 after 5 days treatment with vehicle, VXL, IACS-010759, or the combination of IACS-010759 and VXL (n=3 per treatment);

c) Spleen weight measured after day 5 of treatment with vehicle, VXL, IACS-010759, or the combination of VXL and IACS-010759 (mean±SD, n=4 mice /treatment condition); P-values: **=0.0012; ***=0.0007; ****<0.0001, one-way ANOVA.

d) The percentage of circulating leukemia cells in peripheral blood (PB) at day 0 and day 5 of treatment with vehicle, VXL, IACS-010759, or the combination of IACS-010759 and VXL, as measured by flow cytometry (mean±SD, each dot represents blood sample from individual animal); P-values: *=0.03; **=0.005; ****<0.0001, one-way ANOVA.

e) The percentage of leukemia cells in bone marrow (BM) at day 5 of treatment with vehicle, VXL, IACS-010759, or the combination of IACS-010759 and VXL (flow cytometry) (mean±SD, each dot represents bone marrow sample from individual animal); P-values: *=0.0439; **=0.0052; ***=0.0001, one-way ANOVA.

f) Representative images of H&E staining of bone marrow (BM), spleen, and liver tissues from healthy mice and mice engrafted with T-ALL PDX80 treated with vehicle, IACS-010759, VXL, or the combination of IACS-010759 and VXL (n=3 per treatment).
Scale bars represent 100 μm;
