# Supplementary material for: Inhibition of mitochondrial complex I reverses NOTCH1-driven metabolic reprogramming in T-cell acute lymphoblastic leukemia
Source: Nat Commun. 2022 May 19;13:2801. doi: 10.1038/s41467-022-30396-3 (PMC9120040; doi:10.1038/s41467-022-30396-3)
Supplement: Supplementary file 3 — Reporting Summary [file 41467_2022_30396_MOESM3_ESM.pdf]

nature portfolio

Corresponding author(s): Marina Konopleva

Last updated by author(s): 03/11/2022

Reporting Summary

Nature Portfolio wishes to improve the reproducibility of the work that we publish. This form provides structure for consistency and transparency in reporting. For further information on Nature Portfolio policies, see our Editorial Policies and the Editorial Policy Checklist.

Statistics

For all statistical analyses, confirm that the following items are present in the figure legend, table legend, main text, or Methods section.

n/a	Confirmed	
☐	☒	The exact sample size (*n*) for each experimental group/condition, given as a discrete number and unit of measurement
☐	☒	A statement on whether measurements were taken from distinct samples or whether the same sample was measured repeatedly
☐	☒	The statistical test(s) used AND whether they are one- or two-sided<br>*Only common tests should be described solely by name; describe more complex techniques in the Methods section.*
☐	☒	A description of all covariates tested
☐	☒	A description of any assumptions or corrections, such as tests of normality and adjustment for multiple comparisons
☐	☒	A full description of the statistical parameters including central tendency (e.g. means) or other basic estimates (e.g. regression coefficient) AND variation (e.g. standard deviation) or associated estimates of uncertainty (e.g. confidence intervals)
☐	☒	For null hypothesis testing, the test statistic (e.g. *F*, *t*, *r*) with confidence intervals, effect sizes, degrees of freedom and *P* value noted<br>*Give P values as exact values whenever suitable.*
☒	☐	For Bayesian analysis, information on the choice of priors and Markov chain Monte Carlo settings
☒	☐	For hierarchical and complex designs, identification of the appropriate level for tests and full reporting of outcomes
☒	☐	Estimates of effect sizes (e.g. Cohen's *d*, Pearson's *r*), indicating how they were calculated

Our web collection on statistics for biologists contains articles on many of the points above.

Software and code

Policy information about availability of computer code

Data collection	1) For RNA sequencing<br>FAST QC obtained quality metrics on each FACSTQ input file<br>Kallisto v0.44.059 was used to for pseudoaligning the raw sequencing reads.<br>Abundance data was further analyzed with Sleuth v0.30.060 using models with covariates for condition.<br>The RNA-seq data were further used for NetBID analysis.<br><br>2) For analysis of Genome-Wide Chromatin Occupancy by Notch1, differentially expressed genes for GSEA and heatmaps (http://bioinformatics.sdstate.edu/idep/, https://www.gsea-msigdb.org/gsea/index.jsp) were performed.<br><br>3) For evaluation of synergy effects COMBENEFIT software 2.021 was used.<br><br>4) For evaluation of Seahorse Experiments, Wave software 2.6.1 and Seahorse generators (AGILENT) were used.<br><br>5) RNA Variant Calling Pipeline was used to detect short variants in RNA-seq: we ran the GATK RNA-seq variant calling pipeline (https://gatk.broadinstitute.org/hc/en-us/articles/360035531192-RNAseq-short-variant-discovery-SNPs-Indels-).<br>Quality control and data preprocessing were done with fastp (version 0.20.0).<br>STAR aligner (version 2.6.0b) in two-pass mode aligned to the reference genome b37 (Homo_sapiens_assembly19.fasta).<br><br>6) Metabolomics on cell lines and spleen and BM from in vivo studies was collecting on Hybrid quadrupole-Orbitrap mass spectrometer (Q Exactive, Thermo Scientific) with a Thermo Scientific Accela 1250 ultra-high-performance LC system with an electrospray ionization source, simultaneously operating in positive/negative polarity-switching ionization mode. The LC-MS platform was controlled by XCalibur 2.2 software (Thermo Scientific).

7) Metabolomics on murine blood were acquired using a Thermo Orbitrap Fusion Tribrid Mass Spectrometer under electrospray ionization negative ionization mode and analysed on Inguenity Pathway Analysis (IPA) software (QIAGEN)

9) CYTOF:
Samples were acquired on a Helios CyTOF machine (DVS Sciences) using the Helios 6.5.358 acquisition software (Fludigim).
Individual mass cytometry data files (.fcs) were then filtered using FlowJo to remove the normalization beads, debris, doublets, and dead cells.
The remaining analysis was performed in R (version 3.6.1, The R Foundation for Statistical Computing) using the R packages 'cytofkit' and 'flowcore'.
Processed data were subjected to negative value pruned inverse hyperbolic sine transformation and clustered based on the PhenoGraph algorithm (k=22) using all cell surface markers.
Dimensionality reduction was performed using the uniform manifold approximation and projection (UMAP) method.

10) Western blotting:
The protein signal was visualized using a LI-COR Odyssey imaging system.

Data analysis

1) RNA seq data :
Gene-level abundance estimates were calculated as the sum of transcripts per million (TPM) mapped to a given gene.
The data have been deposited in the NCBI's Gene Expression Omnibus (GEO) database (GSE167305)

2) For NetBID analysis:
TPM-normalized count matrices were log2 transformed and filtered for genes that were not detected in all samples. To reduce the noise introduced by genes with low counts, we used an interquartile range method to rank the genes and selected the top 50% of the highly variable genes to run NetBID analysis on each cell line independently. Next, we merged all the gene sets from the Molecular Signatures Database (MsigDB) and calculated pathway activity in each sample using the mean expression level of genes in the gene sets; we then performed differential activity analysis at the pathway level. Significant pathways were selected at $p < 0.05$ or $p < 0.1$.

3) RNA Vriant Calling Pipeline: Duplicate reads were marked and removed by Picard MarkDuplicates (version 2.9.0). GATK (version 3.7) SplitNCigarReads was used to split and trim reads to remove any overhangs into introns.
Base quality recalibration was performed with GATK (version 4.1.0.0), BaseRecalibrator, and APPlyBQSR.
To correctly handle the splice junctions, the GATK Haplotypecaller was used to perform variant calling with a minimum phred-scaled confidence threshold of 20.0.
The known single-nucleotide polymorphisms were removed using dbSNP (version 138).
Variant call quality was filtered based on Fisher strand values greater than 30.0 and Qual by Depth values below 2.0 with GATK Variant Filtration. To annotate gene variants, we used ANNOVAR and combined each output table.

4) DepMAP Crispr Analysis
Genome-scale CRISPR-Cas9 screen result was obtained at the DepMap Portal (33T33Thttps://depmap.org/portal/achilles33T) with all data (raw read counts to processed gene effect scores) available in the 19q4 DepMap dataset (FigShare: https://figshare.com/articles/dataset/DepMap_19Q4_Public/11384241/3)

5) All raw MS datasets were processed using Sieve 2.2 (Thermo Fisher Scientific), and features with a coefficient of variation lower than 25% in the quality control samples were considered for further analysis. Peaks were scaled according to probabilistic quotient normalization, and features were then mined against an in-house database of accurate masses and retention times generated in our laboratory using the IROA 300 Mass Spectrometry Metabolite Library of Standards (IROA Technologies).

6) Metabolomics on murine blood were analyzed by Thermo Trace Finder software and further processed by Ingenuity pathway Analyze r(IPA) QIAGEN.

7) CYTOF:
Data were first demultiplexed by Fluidigm Debarcoder software.

8) Histology results were analyzed by Aperio Image Scope 12.4.0.5043 software.

9) For evaluation of synergy effects COMBENEFIT software 2.021 was used.

10) Flow cytometry analysis:
Was performed with FlowJo software ver. 10.6.2

11) Data analysis and calculations of statistics:
The data analysis was performed using GraphPad Prism Ver. 9

12) Western-blotting signal was analysed by Image Studio Lite Ver 5.2

For manuscripts utilizing custom algorithms or software that are central to the research but not yet described in published literature, software must be made available to editors and reviewers. We strongly encourage code deposition in a community repository (e.g. GitHub). See the Nature Portfolio guidelines for submitting code & software for further information.

Data

Policy information about availability of data

All manuscripts must include a data availability statement. This statement should provide the following information, where applicable:

- Accession codes, unique identifiers, or web links for publicly available datasets
- A description of any restrictions on data availability
- For clinical datasets or third party data, please ensure that the statement adheres to our policy

in the study following accession codes were used:
GEO-GSE12948,
All requests for raw data and materials will be reviewed by MD Anderson Cancer Center to verify if the request is subject to any intellectual property or confidentiality obligations.
RNAseq data were stored under the accession number GSE167305 and was released
Any data and materials that can be shared by the corresponding author will be released freely or via a Material Transfer Agreement if deemed necessary.

Field-specific reporting

Please select the one below that is the best fit for your research. If you are not sure, read the appropriate sections before making your selection.

☒ Life sciences ☐ Behavioural & social sciences ☐ Ecological, evolutionary & environmental sciences

For a reference copy of the document with all sections, see nature.com/documents/nr-reporting-summary-flat.pdf

Life sciences study design

All studies must disclose on these points even when the disclosure is negative.

Sample size	Since this is a preclinical study, no sample size calculation was performed. Sample size for biological replicates was always more or equal 3. Sample size for RNA seq was to be 2 and this was sufficient to make sound conclusion from data analysis, the choice of samples were done in correlation with flow cytometry data conducted on same material.
Data exclusions	No data was excluded from the analysis.
Replication	All reported data were in the figures were reproducible and were replicated before inclusion.
Randomization	In our preclinical mice studies, all mice were randomized according to the entgraftment level prior experimental treatment administration, here the aim was to allocate mice to each group so that the average of initial engraftment level is equal, comparable between groups, no other randomization procedure was performed since its not a clinical trial study .
Blinding	Blinding was not relevant to our study, as this is preclinical study animal study with no patient subjects participation.

Reporting for specific materials, systems and methods

We require information from authors about some types of materials, experimental systems and methods used in many studies. Here, indicate whether each material, system or method listed is relevant to your study. If you are not sure if a list item applies to your research, read the appropriate section before selecting a response.

Materials & experimental systems

n/a	Involved in the study	
☐	☒	Antibodies
☐	☒	Eukaryotic cell lines
☒	☐	Palaeontology and archaeology
☐	☒	Animals and other organisms
☐	☒	Human research participants
☒	☐	Clinical data
☒	☐	Dual use research of concern

Methods

n/a	Involved in the study	
☒	☐	ChIP-seq
☐	☒	Flow cytometry
☒	☐	MRI-based neuroimaging

Antibodies

Antibodies used	1) The following antibodies were used in the study for CYTOF and was validated by flow cytometry prior study by FLOW CYTOMETRY and CYTOF core facility:<br>127I S-phase Sigma I7125-5G, 139La CD7 CD7-6B7 BioLegend 343102, 142Nd Caspase 3, cleaved D3E9 DVS-Sunnyvale 3142004A,

143Nd PARP, cleaved F21-852 DVS-Fluidigm 3143011A, 144Nd p-AKT M89-61 BD 560397, 147Sm p-STAT5(Y694) 47 DVS-Fluidigm 3147012A, 148Nd CD34 581 BD 555820, 149Sm p-4EBP1 236B4 DVS-Fluidigm 3149005A, 150Nd β-catenin, active 8E7 EMD 05-665, 152Sm p-H2AX, p-γ-H2AX N1-431 BD 560443, 153Eu Notch-1 MHN1-519 BioLegend 352102, 154Sm p21, WAF1/Cip1 CP74 Sigma P1484, 156Gd P-p38 (180/182) D3F9 DVS-Fluidigm 3156002A, 159Tb p-Histone H3 HTA28 BioLegend 641002, 160Gd p-PI3K (p85/p55) Polyclonal CST 4228BF, 161Dy Cytochrome C 7H8.2C12 Abcam ab13575, 162Dy mTOR Polyclonal GenScript A01154, 163Dy c-Myc D84C12 CST 5605BF, 164Dy p-AMPKα (T172) 40H9 CST 2535BF, 165Ho HIF-1α Polyclonal Novus NB100-479, 167Er p-ERK1/2 D13.14.4E DVS-Sunnyvale 3167005A, 168Er Ki67 (Ki67) X8 DVS-Fluidigm 3168001B, 170Er CD3 UCHT1 BioLegend 300443, 174Yb CD5 L17F12 BioLegend 364002, 175Lu p-S6 N7-548 DVS-Fluidigm 3175009A, 176Yb Cyclin B1 GNS-1 BD 554177, 195Pt Cisplatin (dead cells) Sigma P4394-25MG, 89Y CD45 89Y HI30 DVS-Fluidigm 3089003B

2) The following antibodies were used in the study for Western blot analysis:
Notch1 FL 3608s rabbit Cell Signaling 1:1000, Notch1 cleaved 4147s rabbit Cell Signaling 1:1000, p-Akt s473 4060s rabbit Cell Signaling 1:1000, tAkt 2920s mouse Cell Signaling 1:2000, p-4EBP1 s65 9456 s rabbit Cell Signaling 1:1000, 4EBP1 9452s rabbit Cell Signaling 1:1000, p-eIF4E s209 9741s rabbit Cell Signaling 1:1000, eIF4E 9724s rabbit Cell Signaling 1:1000, p-S6 ser240/244 2215s rabbit Cell Signaling 1:1000, S6 2217s rabbit Cell Signaling 1:1000, p-PFKFB2 ser483 13064s rabbit Cell Signaling 1:1000, PFKFB 13045s rabbit Cell Signaling 1:1000, p-LDHA tyr10 8176s rabbit Cell Signaling 1:1000, LDHA 2012 s rabbit Cell Signaling 1:1000, Pyruvate dehydrogenase 3205s rabbit Cell Signaling 1:1000, pLKB1 s428 3482s rabbit Cell Signaling 1:1000, pAMPK t172 2535s rabbit Cell Signaling 1:1000, AMPK 5832s rabbit Cell Signaling 1:1000, pULK s555 5869s rabbit Cell Signaling 1:1000, ULK1 4776s rabbit Cell Signaling 1:1000,
LC3A/B 4108s rabbit Cell Signaling 1:1000, pH2AX s139 2577l rabbit Cell Signaling 1:1000, H2AX 7631s rabbit Cell Signaling 1:000, c-myc 9402s rabbit Cell Signaling 1:1000, pp-S6 kinase t389 97596s rabbit Cell Signaling 1:1000, p-70 S6 kinase 9202 rabbit Cell Signaling 1:1000, Cleaved PARP 5625s rabbit Cell Signaling 1:1000, PARP 9542s rabbit Cell Signaling 1:1000, Cleaved caspase 3 9664L rabbit Cell Signaling 1:1000, Caspase 3 9662s rabbit Cell Signaling 1:1000, LKB1 ab15095 mouse Abcam 1:1000, mtTFA ab47517 rabbit Abcam 1:1000, VDAC1 ab15895 rabbit Abcam 1:1000, Total oxphos human wb ab110411 mouse Abcam 1:1000, GAC ab156876 rabbit Abcam 1:1000, GAPDH ab8245 mouse Abcam 1:5000, Tomm20 ab186735 mouse Abcam 1:2000, α-tubulin ab7291 mouse Abcam 1:2000, β-Actin A5441 mouse SIGMA 1:5000;
Odyssey Irdye 680 RD anti-mouse 1:15000, Odyssey Irdye 800CW anti-rabbit 1:15000 (secondary antibodies).

3) The following flow cytometry antibodies were used in the animal study for engraftment detection and in niche based assay and apoptosis assay:
human CD45 FITC BIOLEGEND 1 ul/100ul, mice CD45 APC BIOLEGEND 1 ul/100ul; Annexin V APC BD BIOSCIENCE 1:100; murine anti-Thy1.2 PE BD BIOSCIENCE 1 ul /100 ul, murine anti-CD4 PE Cy5 BD BIOSCIENCE 1 ul/100ul, murine anti-CD8 APC BD BIOSCIENCE 1ul/100ul, murine anti-CD25 PE-Cy7 BD BIOSCIENCE 1 ul/100ul and murine anti-CD44 AF780 BD BIOSCIENCE1 ul/100 ul

Validation

The above listed antibodies used for flow cytometry were validated and titrated by flow cytometry.
The CyTOF antibodies were validated by flow cytometry by Flow Cytometry and Cellular Imaging Core Facility at MDACC.

Eukaryotic cell lines

Policy information about cell lines

Cell line source(s)

The T-ALL cell lines: JURKAT, PF-382, 1301, TALL-1, LOUCY, P12-ICHIKAWA, MOLT-3, MOLT-4, CCRF-CEM, SUPT1 and KOPT-K1 were obtained initially from ATCC were maintained at an internal core facility of The Institute of Science in Cancer MDACC and authenticated by short tandem repeat DNA fingerprinting in February 2016.
MOLT-16 (ACC 29), DND-41 (ACC 525), ALL-SIL (ACC 511), and HPB-ALL (ACC 483), were purchased from DSMZ in March 2019.
MS5 (ACC 441) were obtained initially from DSMZ and fMS5 DL4 cell line was generated in Dr Trang Hoang lab.

Murine T-ALL cells were generated and obtained from Dr. D. Herranz and Dr. A. Ferrando using initially the following mice strain: Rosa26Cre-ERT2/+ Gls f/f . This murine T-ALL cell model was expanded in C57BL6 mice.

Authentication

All cell lines were authenticated by short tandem repeat DNA fingerprinting performed by an internal core facility of The Institute of Science in Cancer MDACC.

Mycoplasma contamination

All cell lines were subjected to mycoplasma testing and were negative for mycoplasma.

Commonly misidentified lines
(See ICLAC register)

No misidentified lines were used in this study.

Animals and other organisms

Policy information about studies involving animals; ARRIVE guidelines recommended for reporting animal research

Laboratory animals

For the studies of pre-LSCs, all mouse lines were backcrossed onto a C57BL/6J background for more than 12 generations as previously described to produce pSil-TSCL (SCLtg), Lck-LMO1 (LMO1tg), and Lck-NotchIC9 (Notch1tg) mice (NIAID/Taconic Repository). Mice were maintained in pathogen-free conditions according to institutional animal care and use guidelines set by the Canadian Council on Animal Care.

For the studies of T-ALL, all experimental animal procedures were approved by MD Anderson Cancer Center's Institutional Animal Care and Use Committee (IACUC). The study was compliant with all relevant ethical regulations regarding animal research. Animal studies were conducted at MD Anderson's animal facilities in accordance with the IACUC guidelines. Eight-week-old 57BL/6 female mice and 8- to 10-week-old female NOD Cg-PrkdcscidIl2rgtm1Wjl/SzJ (NSG) mice were purchased from The Jackson Laboratory. Mice were maintained in a pathogen-free environment with free access to food.

Murine T-ALL cells were generated and obtained from Dr. D. Herranz and Dr. A. Ferrando using initially the following mice strain: Rosa26Cre-ERT2/+ Glsf/f this mice model was expanded in C57BL6 mice.
NSG mice were used to conduct all studies with human PDX models.

Wild animals	No wild animals were used in the study.
Field-collected samples	No field collected samples were used in the study.
Ethics oversight	All animal studies for preleukemic model examination were performed as per approval of institutional animal care of University of Montreal. The efficacy study in T-ALL were conducted at MDACC, as per approval by IACUC, protocol number 00001146-RN02.

Note that full information on the approval of the study protocol must also be provided in the manuscript.

Human research participants

Policy information about studies involving human research participants

Population characteristics	Patient samples specimen was collected during standard diagnostic procedures after informed consent was obtained in accordance with the Institutional Review Board (IRB) regulations of MD Anderson Cancer Center.
Recruitment	*Describe how participants were recruited. Outline any potential self-selection bias or other biases that may be present and how these are likely to impact results.*
Ethics oversight	Patient samples were collected under IRB protocol PA13-1025

Note that full information on the approval of the study protocol must also be provided in the manuscript.

Flow Cytometry

Plots

Confirm that:

- [x] The axis labels state the marker and fluorochrome used (e.g. CD4-FITC).
- [x] The axis scales are clearly visible. Include numbers along axes only for bottom left plot of group (a 'group' is an analysis of identical markers).
- [x] All plots are contour plots with outliers or pseudocolor plots.
- [x] A numerical value for number of cells or percentage (with statistics) is provided.

Methodology

Sample preparation	The experiments evaluating effects of IACS-010759, CB-839 or VXL in combination were performed on cell lines, leukemic blast enriched from patient samples, T-lymphocytes enriched from buffy coats by CD3+ magnetic beads selection kit or on freshly harvested healthy bone marrow from donors. Further knockdown studies were performed on cell lines transduced with GLS KD lentivirus construct and further enriched by culturing cells in media containing puromycin.<br>Finally purified CD4-CD8- thymocytes isolated from 5-6-week-old preleukemic transgenic mice LMO1tg were co-cultured on MS5 or MS5-DL4.
Instrument	Beckman Coulter flow cytometer and LSRII flow cytometer
Software	Beckam Coulter KALUZA, FlowJo 10.6; FACS Celesta
Cell population abundance	The experiments were performed :<br>on cell lines either parental or modified by GLS KD<br>on patient samples/PDX depleted from CD3+ cells<br>on lymphocytes derived from buffy coats of healthy donors enriched for CD3+ cells<br>on murine CD4−CD8− thymocytes<br>on healthy donor Bone marrow cells
Gating strategy	For apoptosis studies of T-ALL cell lines and patient samples:<br>SSCA/FSCA gating was used for cell gating, FSC-A/FSC-H was used to determine singlets, Annexin APC/ DAPI was used for determination of apoptotic cells and viable cells, histogram from FSC-H/DAPI negative cells were used for ROS evaluation.<br>For viability evaluation in preleukemic models:<br>SSCA/FSCA gating was used for cell gating, FSC-A/FSC-H was used to determine singlets, further gating on PE–anti-Thy1.2, PE-Cy5–anti-CD4, APC–anti-CD8, PE-Cy7–anti-CD25, and AF780–anti-CD44, was performed.

For animal studies:
in NSG mice: SSCA/FSCA gating was used for cell gating, FSC-A/FSC-H was used to determine singlets, FSC-H/DAPI was used to determine viable cells, mice APC/ human FITC was used for determine the percentage of human engraftment in NSG mice.
in C57/BL6 mice: SSCA/FSCA gating was used for cell gating, FSC-A/FSC-H was used to determine singlets, FSC-H/DAPI was used to determine viable cells, mice APC/GFP gating was utilized to determine engraftment of murine leukemic cells.

☒ Tick this box to confirm that a figure exemplifying the gating strategy is provided in the Supplementary Information.
